# Shaped stone balls were used for bone marrow extraction at Lower Paleolithic Qesem Cave, Israel

**DOI:** 10.1371/journal.pone.0230972

**Published:** 2020-04-09

**Authors:** Ella Assaf, Isabella Caricola, Avi Gopher, Jordi Rosell, Ruth Blasco, Oded Bar, Ezra Zilberman, Cristina Lemorini, Javier Baena, Ran Barkai, Emanuela Cristiani

**Affiliations:** 1 Institute of Archaeology, Tel-Aviv University, Tel-Aviv, Israel; 2 DANTE—Diet and Ancient Technology Laboratory, Department of Oral and Maxillofacial Sciences, Sapienza University of Rome (IT), Rome, Italy; 3 Newcastle University, School of History, Classics and Archaeology, Newcastle Upon Tyne, England, United Kingdom; 4 IPHES Institut Català de Palaeoecologia Humana i Evolució Social,Tarragona, Spain; 5 Àrea de Prehistòria Universitat Rovira i Virgili (URV),Tarragona, Spain; 6 Centro Nacional de Investigación sobre la Evolución Humana (CENIEH), Burgos, Spain; 7 Geological Survey of Israel, Jerusalem, Israel; 8 Department of Classics, LTFAPA Lab., Sapienza University of Rome (IT), Rome, Italy; 9 Department of Prehistory and Archaeology, Universidad Autónoma, Madrid, Spain; Universita degli Studi di Ferrara, ITALY

## Abstract

The presence of shaped stone balls at early Paleolithic sites has attracted scholarly attention since the pioneering work of the Leakeys in Olduvai, Tanzania. Despite the persistent presence of these items in the archaeological record over a period of two million years, their function is still debated. We present new results from Middle Pleistocene Qesem Cave on the use of these implements as percussion tools. Use-wear and abundant bone and fat residues found on ten shaped stone balls indicate crushing of fresh bones by thrusting percussion and provide direct evidence for the use of these items to access bone marrow of animal prey at this site. Two experiments conducted to investigate and verify functional aspects proved Qesem Cave shaped stone balls are efficient for bone processing and provide a comfortable grip and useful active areas for repeated use. Notably, the patina observed on the analyzed items precedes their use at the cave, indicating that they were collected by Qesem inhabitants, most probably from older Lower Paleolithic Acheulian sites. Thus, our results refer only to the final phases of the life of the items, and we cannot attest to their original function. As bone marrow played a central role in human nutrition in the Lower Paleolithic, and our experimental results show that the morphology and characteristics of shaped stone ball replicas are well-suited for the extraction of bone marrow, we suggest that these features might have been the reason for their collection and use at Qesem Cave. These results shed light on the function of shaped stone balls and are consistent with the significance of animal fat in the caloric intake of Middle Pleistocene humans as shown by the archeozoological evidence at Qesem Cave and possibly beyond.

## Introduction

Shaped stone balls (henceforth SSBs) are a remarkable component at sites of the Oldowan and Acheulian cultural complexes in Africa [1–8], Asia [9–11], and Europe [12], as well as at Middle Stone-Age African sites [13]. Despite their conspicuous and prolonged presence, and the intensive scientific research focused on them, their typological definition and function are still debated [14–23]. Kleindienst [15] proposed a division into three categories based on type and degree of skills required for their manufacture: Missiles (roughly spherical, mostly shaped by nature but also showing signs of intentional shaping); polyhedral (roughly spherical, shaped by faceted intersecting negative flake scars over most of their surface or their entire surface); and bolas (pecked and/or battered to a nearly smooth surface and nearly spherical). Leakey [16] suggested (based on the SSBs found at Olduvai) a subdivision to polyhedrons (*“…angular tools with three or more working edges*, *usually intersecting”)*, spheroids (*“…stone balls*, *smoothly rounded over the whole exterior*. *Faceted specimens in which the projecting ridges remain or have been only partly removed are more numerous…”)*, and sub-spheroids (*“…similar to the spheroid but less symmetrical and more angular…”*). Leakey’s suggestion was later criticized, and other definitions were proposed. Sahnouni [17], for example, distinguished between two types of SSBs: Polyhedrons, items flaked on at least three different faces and with some relatively acute edges but a fairly obtuse average core angle; and spheroids, items heavily flaked over much or all of the exterior with very obtuse angles. The first of two current opposing views interprets SSBs as end products of a preconceived shaping process [14], used as bolas or throwing stones for capturing animals [18–20] or as food-pounding tools [21]. The second view interprets these items not as predetermined tools but as byproducts of specific technological or functional trajectories: Exhausted cores [17, 22], hammerstones [1, 7, 23], or battering tools for processing vegetal material or tendering meat [2]. However, no conclusive arguments about their purpose have been presented, and their function remains ambiguous.

The presence of SSBs at Qesem Cave, Israel (420–200ka) marks the latest appearance of this type of artifacts in the Lower Paleolithic Levant and represents the end of a long tradition of over two million years of producing and using SSBs. A residue and use-wear study yielded significant data regarding ten well-preserved SSBs (out of a total of twenty-nine specimens analyzed) from Qesem Cave, shedding new light on the use of these items. While the original function of these tools is impossible to study due to the cover of patina, we demonstrate that the cave inhabitants selected and collected specifically old, patinated SSBs from outside the cave and then used them in thrusting percussion actions for crushing fresh animal bones to access the marrow. Collecting artifacts produced elsewhere and bringing them to the cave was a behavioral pattern familiar to the Qesem inhabitants. A plethora of evidence shows that they regularly selected, collected, and transported older (sometimes patinated) lithic blanks, which were then used, sometimes in a manner of recycling, inside the cave [24–30]. Several studies exploring the presence of patinated items at Qesem Cave (including analysis at low and high magnification using stereomicroscope and metallographic microscopes) demonstrated that the original surface of many items was affected by a medium-heavy glossy appearance and an alteration typical of prolonged exposure to the elements in open environments. The patinated, knapped blanks and shaped tools from outside of the cave [see 25, 31] amount to the 10% of all the analyzed lithic assemblages [24]. This argument is based on the following observations: At Qesem Cave, patinated flaked items are present alongside a majority of “fresh” unpatinated items in the same contexts throughout the 11 m of stratigraphic column. Moreover, among the patinated items, only a few were not recycled. It would have been expected to find more unrecycled patinated items had the patina formed on-site. The variation of colors and textures of the patinated surfaces is high, and therefore unlikely to have been formed on-site [24]. In contrast, patina that was noted to have been formed inside the cave is homogenous, characterized by a light, translucent white color that cannot usually be observed by the naked eye [25]. Further on, we suggest that collecting these 'older' previously knapped artefacts was a behavioral pattern practiced by the cave's inhabitants. These artefacts include scrapers and bifaces that were brought to the cave in their current state (covered with patina, rather than being produced inside the cave). More specifically, and similarly to the SSBs, it had been recently argued based on flint type analysis that the handaxes found on site were actually collected from older contexts, most likely Acheulian sites existing in the vicinity of the cave, and brought to the cave as readymade objects [32]. We suggest that also the SSBs described in this study are part of the behavioral pattern of collecting old artefacts practiced by the cave's inhabitants. It seems that these items were produced elsewhere, then some of them split into halves, either as a technological result of their shaping or following heavy-duty use, and patina accumulated on them. The formation of use-wear signs and residue above the patinated surfaces suggests the analyzed SSBs (complete, broken and/or patinated) were collected, brought to the cave and used inside.

Distinct use-wear traces and residues observed on SSBs found at Qesem Cave associate them with bone-breaking activities. Still, a question should be raised: If this is the case, why were these specific items chosen for such particular activity? In this paper we will discuss the results of the functional analysis and suggest, based on archaeological data and experimental work, that the SSBs were indeed selected by the cave’s inhabitants due to their distinctive man-made morphological characteristics, which were best suited for marrow extraction tasks. We suggest that SSBs’ are yet another example of the production of a tool with a particular morphology according to the activity (and grasping/handling process) for which it was designed [33–34]—in this case percussive activity. We infer that these items were technologically shaped in this particular form due to their specific role within Lower Paleolithic toolkit as percussion instruments.

## The site

The Middle Pleistocene archaeological site of Qesem Cave is located on the western slopes of the Samaria hills, 12 km east of the Mediterranean. Various methods suggested dating human occupation at the cave started at ca. 420 ka and ended prior to 200 ka [35–38]. The habitual use of fire is apparent throughout the sequence of the cave by the presence of wood ash and hearths [39–40], as well as by the large amounts of burned flint and bones [41], indicating that activities were organized around the hearth as a center of activity [42].

The site yielded a rich faunal assemblage mostly dominated by bones of fallow deer, supplemented by red deer, horses, aurochs, wild pigs, wild asses, tortoises, and birds [41–44]. Many bones show cut marks, burning damage, and damage caused by bone breakage, indicating that butchering, roasting, and marrow extraction took place at the site [42, 45]. The ungulate mortality profile is dominated by adult individuals, and in the case of fallow deer, the relative abundance of cubs and young individuals suggests seasonal hunting episodes [41–42]. The focus on hunting prime-aged fallow deer (with the highest fat content) and the bias towards higher-utility body parts indicate the importance of fat and marrow in the Qesem hominin transport decisions. Evidence of diagnostic taphonomic elements of bone breakage in previous studies indicates that the marrow of both long and flat bones was accessed through direct percussion [42]. The bone surface modifications resulting from the anthropogenic breakage include percussion pits, notches, impact flakes (cortical flakes and scars included), counterblows, and peeling. Burning damage affects more than 30% of the bone fragments in all assemblages. The presence of burning signs on bone fragments might indicate preparation of bones to facilitate breakage [45–46] or preparation of the marrow for removal [47–48].

The lithic assemblages are characteristic of the Acheulo-Yabrudian Cultural Complex of the late Lower Paleolithic period in the Levant [49]. Blade-dominated Amudian contexts are present throughout the sequence, showing a full *chaîne opératoire* that includes well-selected flint nodules, core shaping, blade production (including blades and retouched blades), use, and discard [50–51]. Quina (and demi-Quina) scraper-dominated Yabrudian contexts appear in three areas of the cave only [52]. Several additional stone-tool reduction strategies were discerned, including the production of flakes and flake tools and a recycling sequence aimed at producing small sharp items from 'older' flakes [28–29]. In addition, bifaces were present on a small scale. However, they seem to have been produced not on site but possibly gathered elsewhere and brought to the cave [32]. Amudian and Yabrudian lithic technologies reflect innovative choices of skilled knappers after the long technological persistency that characterized the Acheulian. Within this context of technological innovations, the presence of SSBs at Qesem Cave stands out as an expression of what we might call anachronism [52]. The intriguing presence of SSBs in this specific cultural context was preliminarily reported and discussed [52]. However the current study presents innovative results of a combined use-wear and residue analysis never conducted nor presented before on SSBs.

## Materials

### The SSB sample: Archaeological contexts

**Fig 1:**
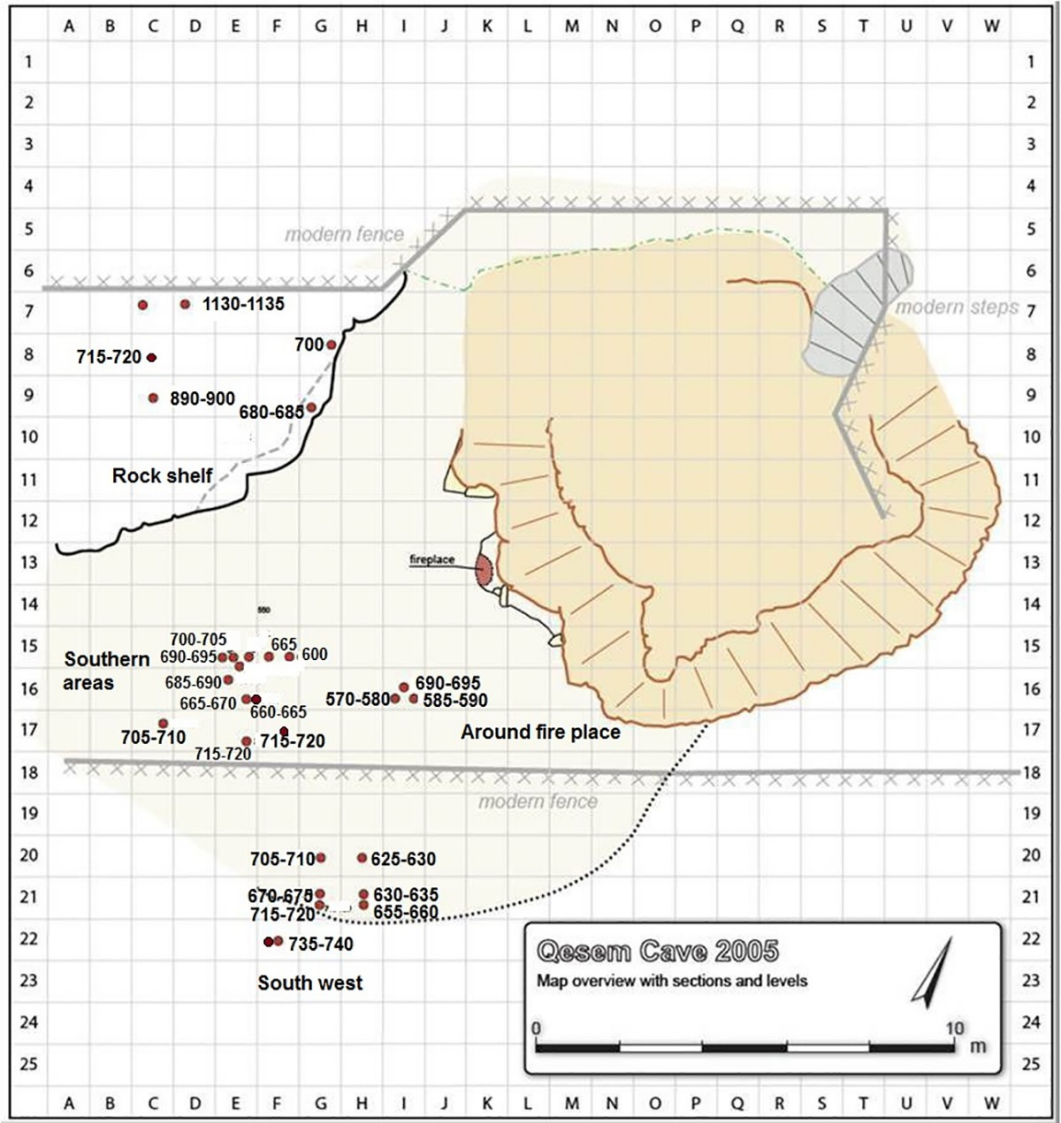
Map of Qesem Cave site. Red circles indicate the location of SSBs: Ten items found in the south west area; six items under the rock shelf; nine items in the southern areas; two items around the fireplace; two items found in non-excavated contexts).

Twenty-nine SSBs found at Qesem Cave include spheroids and sub- spheroids, and also five split half-balls, out of which ten items yielded residue and use-wear traces. The other nineteen tools display neither observable functional traces nor residues accumulated before or after their patination (although we cannot rule out the possibility that these items were used in the cave; it is possible that functional traces were not preserved on these cases as such modifications are rare on ancient artifacts). The SSBs were concentrated in particular Amudian contexts of the lower stratigraphic sequence of the cave [52]. One group of nine items was discovered in the southern part of the cave in an area restricted to about five square meters. Two items were found within a single square meter at similar elevation. Another group, comprising ten items, was discovered in the south western zone of the cave (an area of four square meters): Four stone balls were found within a single square meter, two were found within another one-half square meter, and two within a third one-half square meter, all at similar elevations. Two additional stone balls were found in a one-half square meter area adjacent to the central hearth from the south. Six items were found under the rock shelf in various excavation units and at different elevations, and two were collected from inside the cave within non-excavated contexts when the cave was first discovered (see Fig 1, Table 1, and [52] for further details): Qesem Cave was discovered in 2000 during the construction of a road, after an explosion. Two SSBs included in this study were found in this stage on the surface of the cave, before excavation had begun. Even though these items were not found in a well-defined, excavated context, they were found inside the cave (which was sealed up until that moment).

**Table 1:** Location of SSBs in Qesem Cave.

Context in the cave	No.	Square and elevation below datum	Industry	Traces (items included in the functional study)
**Southwest**	**1**	E15c 690–695	Amudian	
	**2**	E15c 700–705	Amudian	
	**3**	E16a 685–690	Amudian	
	**4**	E16d 665–670	Amudian	
	**5**	E16d 660–665	Amudian	
	**6**	F15c+d 665	Amudian	
	**7**	E17d 715–720	Amudian	Use-wear/residues
	**8**	F15c 715–720	Amudian	Use-wear/residues
	**9**	C17b 705–710	Amudian	
	**10**	F15d 600	Amudian	
**South**	**11**	I16 690–695	Amudian	
	**12**	F22 735–740	Amudian	
	**13**	F22 735–740	Amudian	Use-wear
	**14**	G20 705–710	Amudian	
	**15**	G21 715–720	Amudian	Use-wear
	**16**	H20 625–630	Amudian	
	**17**	H21 655–660	Amudian	Use-wear/residues
	**18**	H21 630–635	Amudian	Use-wear/residues
	**19**	G21 670–675	Amudian	Use-wear/residues
**Rock shelf**	**20**	G9c 680–685	Yabrudian	
	**21**	C9 890–900	Isolated item	Use-wear/residues
	**22**	G8b 700	Amudian	
	**23**	C7a	Amudian	
	**24**	C8 715–720	Amudian	
	**25**	D7a 1130–1135	Yabrudian	
**Around the hearth**	**26**	I16c 570–580	Amudian	Use-wear/residues
	**27**	I16d 585–590	Amudian	
**Non-excavated context inside the cave**	**28**	-	Unknown	Use-wear/residues
	**29**	-	Unknown	

No permits were required for the described study, which complied with all relevant regulations. All specimens described in the manuscript were excavated under permits granted by the Israel Antiquity Authority to RB and AG and are stored at the repository of Institute of Archaeology, Tel-Aviv University. The specimens are available upon request.

### Rock types used in SSB production

The SSBs were made of hard, carbonate rocks (either limestone or dolomite), with the exception of one made of flint. These massive carbonates are different from the highly weathered Turonian limestone that builds the cave area, which is covered by colluvial materials and even some calcrete. Remarkably, whereas the lithic assemblages of Qesem comprise hundreds of thousands of flint items, only a few carbonate rock artifacts (mostly flakes, with the exception of SSBs) were recovered [52]. The absence of carbonate rock waste material raises the question of whether the SSBs were produced on site or collected elsewhere. In addition, a preliminary geological survey (conducted by OB and EZ) indicated that the limestone originating from the natural formation of the cave itself is of low quality, weathered and abraded.

In this study, several SSB replicas were produced (by JB, see below). The reduction strategy required careful planning (see also [14]) and high-quality materials as well as know-how and precision (technological aspects will be further investigated and presented in a different paper). It seems likely that for technological reasons, the stone balls found at Qesem were not shaped from the local, low-quality limestone of the cave’s natural formation, but from nonlocal, higher quality materials. As a matter of fact, non-homogeneous, abraded materials will quickly break during production and may lead to a much smaller final product, suggesting that these shaped items were brought to the cave by its inhabitants from an unknown location outside. As the cave was repeatedly used during a time range of over 200kyr, massive amounts of lithic materials, animal carcasses, and firewood were procured and brought to it regularly [53]. Tools or blanks were also brought to the cave from elsewhere [29, 49, 52], and some of these were recycled inside [24–26, 28–29]. We argue that the preference for previously shaped spherical and subspherical objects will become more coherent in light of the results of our functional analysis presented in this paper.

## Methods

The function of the SSBs was revealed by an integrated use-wear and residue analysis. Methods and criteria for identifying, describing, and interpreting macro- and micro-wear and residues on stone tools were based on well-known literature in the field of functional studies of material culture [8, 54–66]. Use-wear and residues preserved on the SSBs were analyzed at DANTE—*Diet and Ancient Technology Laboratory* (at Sapienza University of Rome) (1) using a digital stereomicroscope (Zeiss AXIO Zoom, with magnification ranging from 10× to 173×) and (2) using a metallographic microscope (Zeiss Axio Scope A1, with magnification ranging from 50× to 500×).

Following the analysis of the surface patinas and the post-depositional modifications affecting the archeological tools, the state of preservation of the residues was analyzed at low and high magnification. Particular care was taken in understanding the stratigraphic relation between the residues and post-depositional concretions, particles of soil as well as functional modifications. Only residues characterized by a strict correlation with the use-wear traces were considered reliable.

Residues were described according to their appearance, using variables such as morphology, texture, color, and birefringency. Possible alteration caused by post-depositional modification and/or mechanical stresses affecting the appearance of the residues and related to use were also documented. Morphological features of animal structures were recorded along with their spatial distribution over the tools [67–69].

The interpretation of archaeological residues was based on the comparison with an experimental macro-residues collection (see below) as well as using the available literature on experimental residues on stone tools [64–65, 70–74].

Functional traces were described following the tribology-based variables proposed by Adams [54]. These variables are grouped in four categories: adhesives (residues), fatigue (fracture, crack, pits and frosted appearance), abrasive (striation, levelling, grain edge rounding) and tribo-chemical wear (polish/sheen).

Following the analysis and documentation of residues on the archaeological tools, SSBs were washed with a 2% solution of demineralized water and neutral phosphate detergent (Derquim®) to allow the correct observation of the micro-traces, sometime hindered by the presence of residues and soil particles adhering the surfaces. As the direct observation of micro-polishes under the metallographic microscope was prevented by the height of the archeological tools as well as by the working distances of the objectives, silicon molds of the areas characterized by use-wear traces were made using Provil Novo Light Fast Heraeus®. Silicon casts were made only on a selection of tools in order not to damage archaeological surfaces (the silicon may stain surfaces), if macro-traces and residues were diagnostic.

A series of experiments was carried out in order to verify archaeological results. In particular, use-wear traces and residues identified on SSBs served as a basis for designing specific experimental activities, which included first replicating and tracing the shaping process of the SSBs, and then testing the SSB replicas in bone crushing activities aimed at marrow extraction. We also used unmodified limestone and dolomite cobbles of different sizes and characteristics in the same experimental activities.

### Functional analysis of the archaeological SSBs

**Fig 2:**
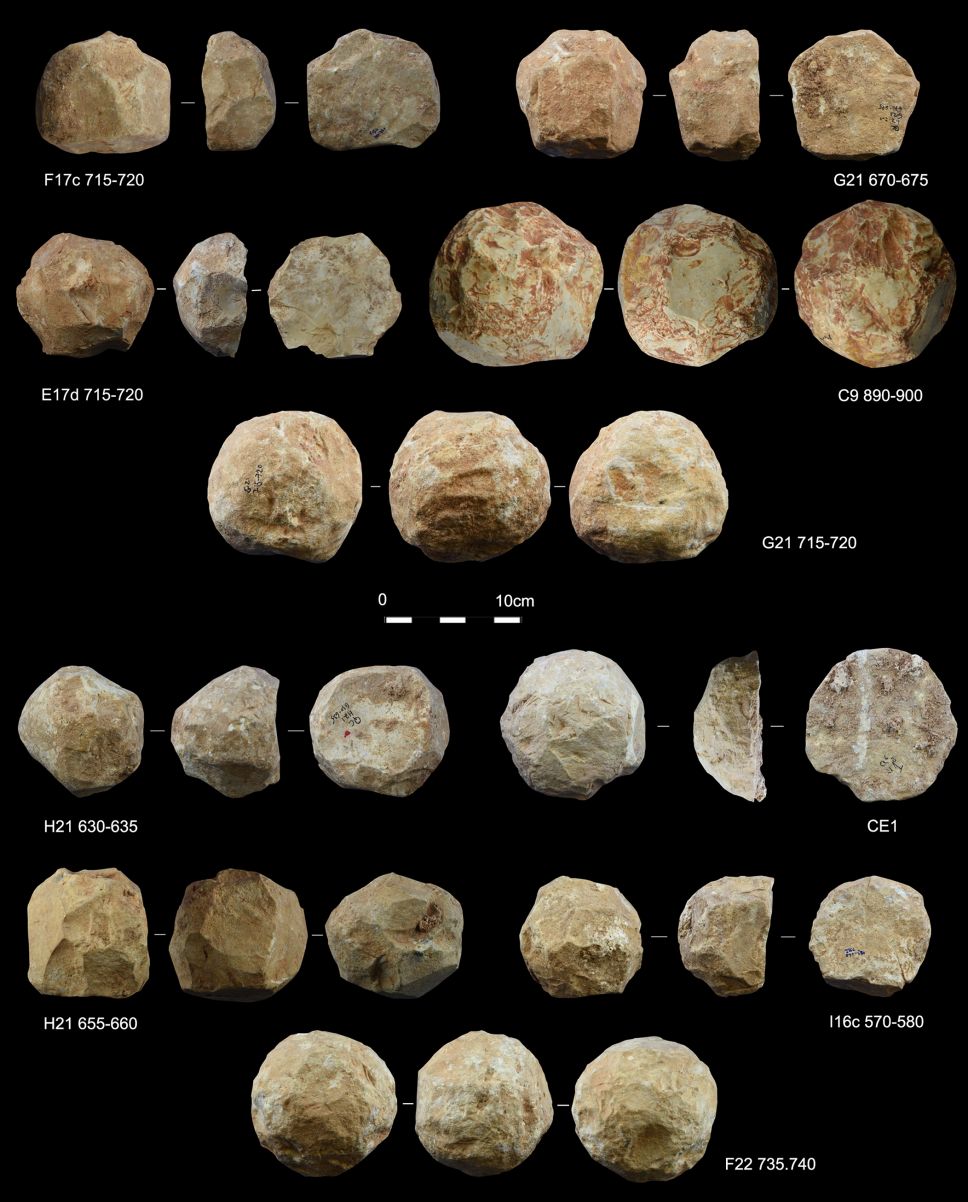
Archaeological sample of SSBs found in Qesem Cave. The archaeological samples discussed in the paper and characterized by use-wear and/or residues. For each specimen, three surfaces are presented (the white line indicates the side of the progressive rotation).

**Fig 3:**
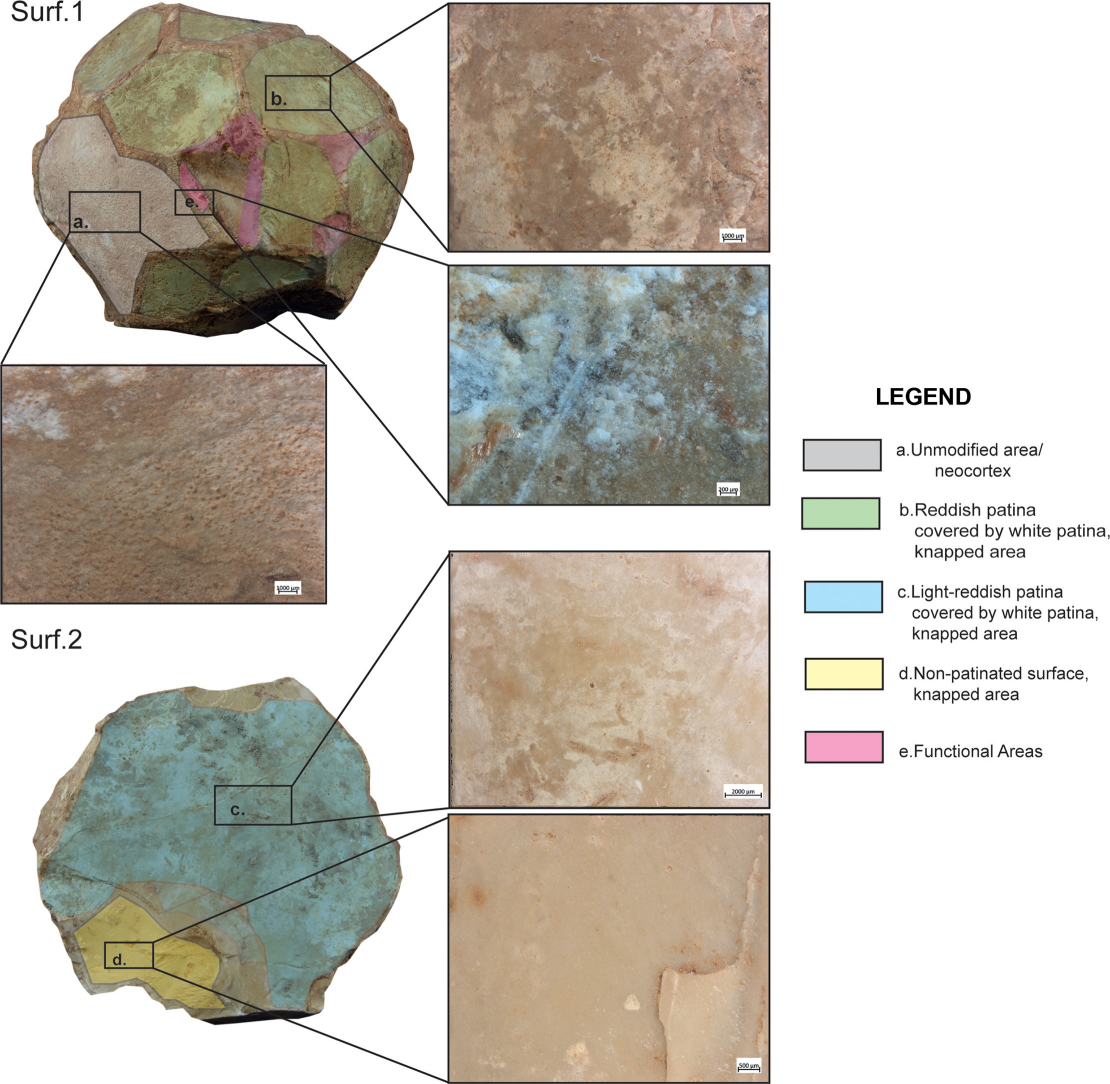
Half-ball from Qesem Cave. Images of patinas with different colors and thicknesses, identified on the two surfaces of the tools. *Surface 1*: (gray) unmodified area with the neocortex; (green) knapped surface with the reddish and white patina; *Surface 2*: (blue) light-reddish patina covering the white patina on the knapped surface; (yellow) non-patinated surface on the knapped area; (pink) localization of the functional areas.

Ten out of twenty-nine items showed developed use-wear traces and/or residues (Fig 2). All SSBs in our sample (including the half-balls) were covered by a patina which preceded their use. The observation of the weathered areas indicated patina formation on top of flaked surfaces, and evidence of use and residue on top of the patina. The patinas show distinctive colors (reddish and white) indicating it originated in different environments [75]. It is important to mention that these objects are almost or totally knapped, and neocortex is preserved only in some unmodified areas of the tools. Traces and residues are found above the patinas hence marking the last and only identifiable cycle of use of the tools marked by the functional traces developed (Fig 3). Detailed studies will be necessary to understand which types of weathering (i.e. deposit and/or dissolution) determined the formation of certain patinas and to infer how many times these objects have been technologically reworked and used. Interestingly, alterations comparable to the ones observed on the SSBs are not typical of other lithic industries found at Qesem cave.

Since use-wear traces and residues overlay on top of the weathered areas of the items, a hypothetical timeline was established for their life history. First came the shaping of the typical ball or polyhedron (and perhaps its original use and discard); next came the development of the surface patina; last, patinated SSBs were collected, brought to the cave, and used (a somewhat similar situation was recently observed at the site of Barranco Leon, [76]). To date, it is impossible to determine any use of the SSBs prior to the patina formation as the development of the latter covered any such evidence. The presence of patinated half-balls, however, might suggest that at least some of these items were used during the initial, pre-patinated phase as their breakage patterns seem to be anthropogenic rather than the result of post-depositional processes (this aspect will be further examined in future studies). In other words, these items were brought to the cave already split and patinated, and then used inside the site.

**Fig 4:**
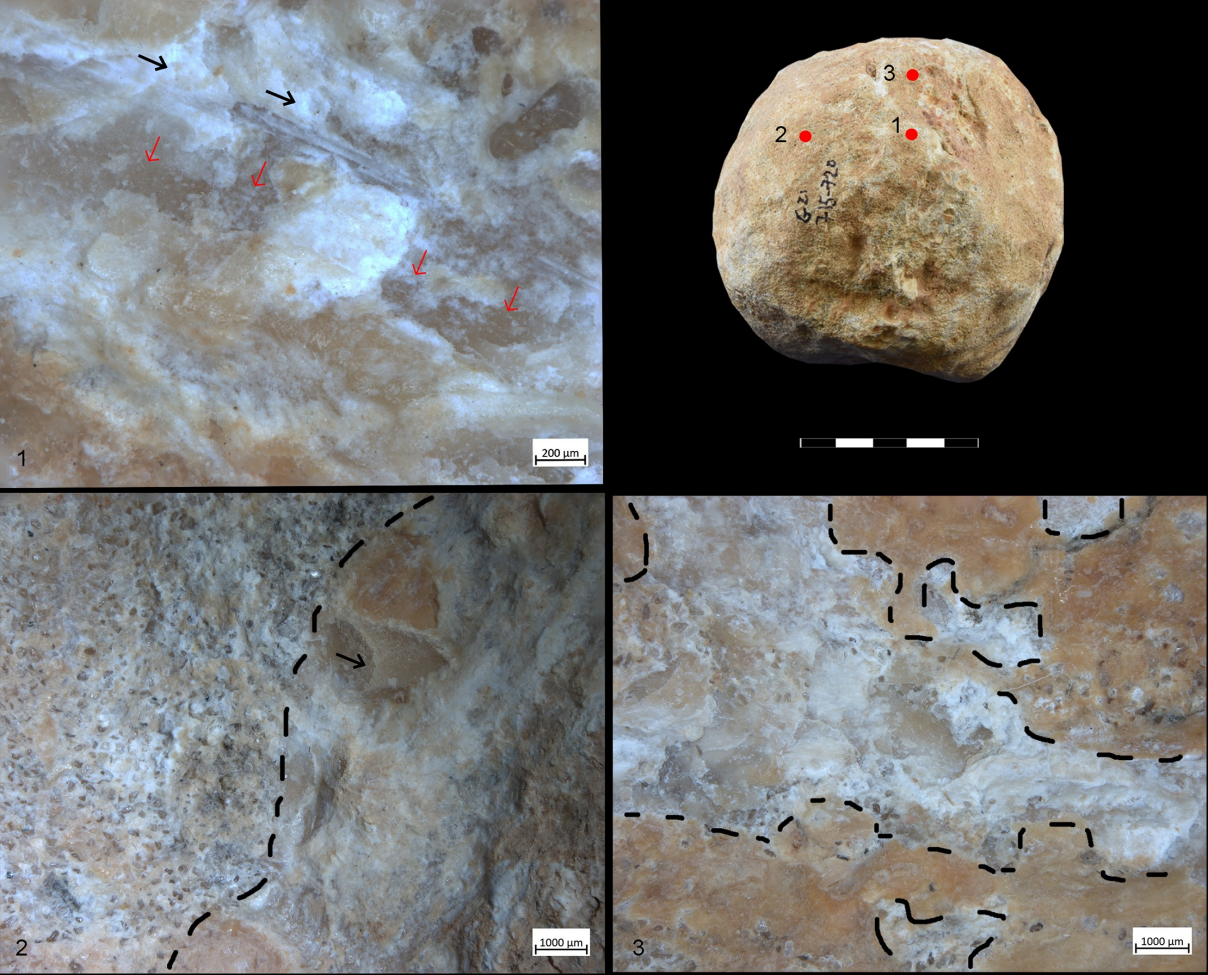
SSB from Qesem Cave (G21 715–720). An example of patina removed by subsequent formation of use-wear. 1) Area with developed traces. Note long oriented striations (see black arrows) associated to negatives of micro-flakes (indicated by red arrows) observed at 50x; 2) Surface of the spheroid showing a granular patina covered by an orange and white patina (the black arrow indicates detachments that removed the patina) documented at 20x; 3) Detail of the patina removed by the use-wear. Note the cracks characterizing the orange and granular patinas observed at 40x.

For all the reasons exposed so far, our use-wear and residue data refer to the most recent use of the SSBs exclusively (Fig 4)

### Archaeological results

**Fig 5:**
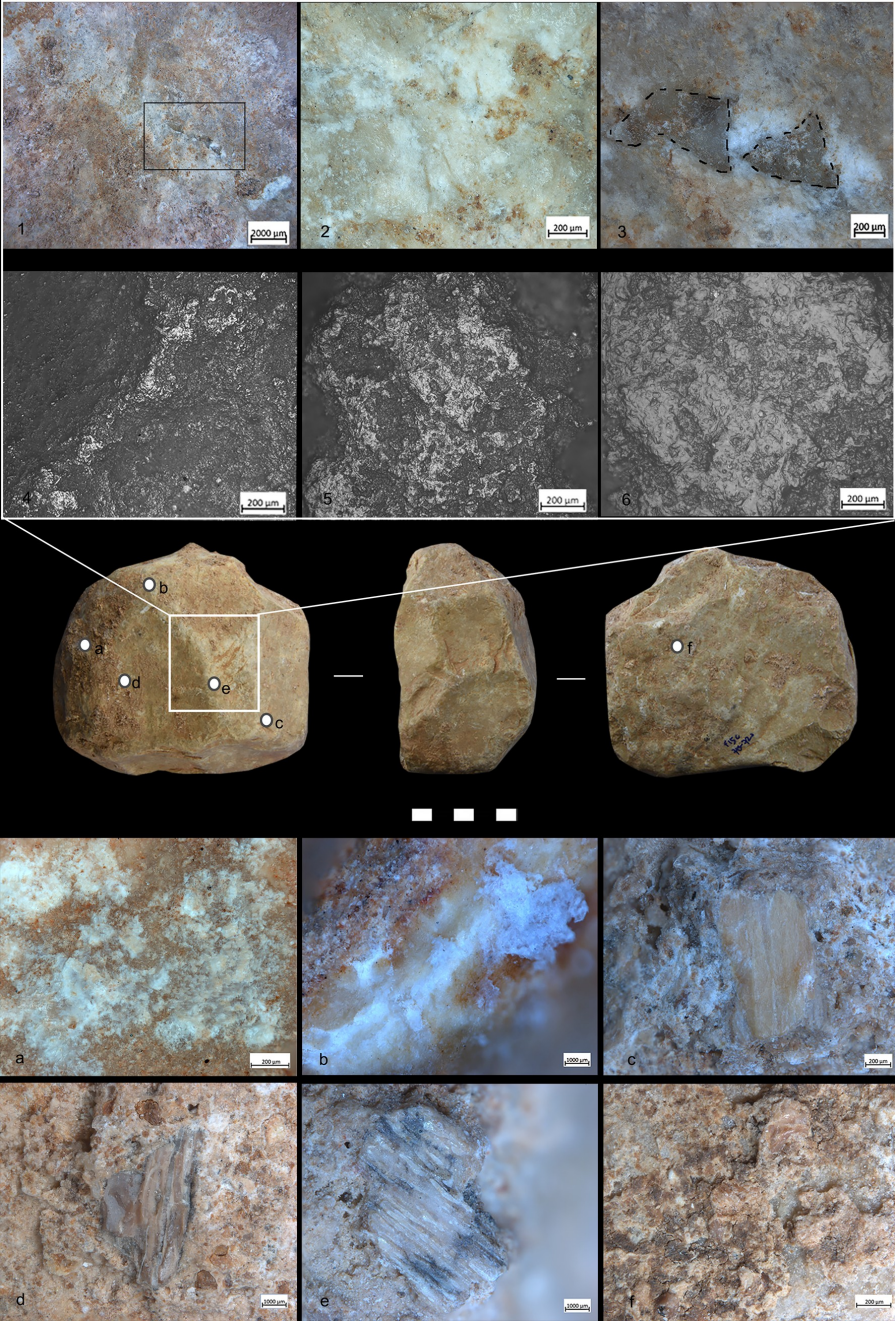
Archaeological SSB (F17c 715–720). *Selection of macro-traces and residues preserved on one archaeological SSB from Qesem Cave*. 1) Negatives of flakes localized on prominent ridges (10×); 2) Sheen surface (20×); 3) Detail of negative flakes (50×); 4 and 5) Micro-polishes localized on high ridges (50×–100×); and 6) Polish with smooth texture and domed topography (200×). The letters (a–f) indicate different types of residues identified on the archaeological tool: a–b) Spots of crushed amorphous white fatty residues and glossy film mixed with bone fiber; c–e) Crushed compact and spongy bone tissues; f) Spots of crushed greasy fat matter mixed with bone fragments.

**Fig 6:**
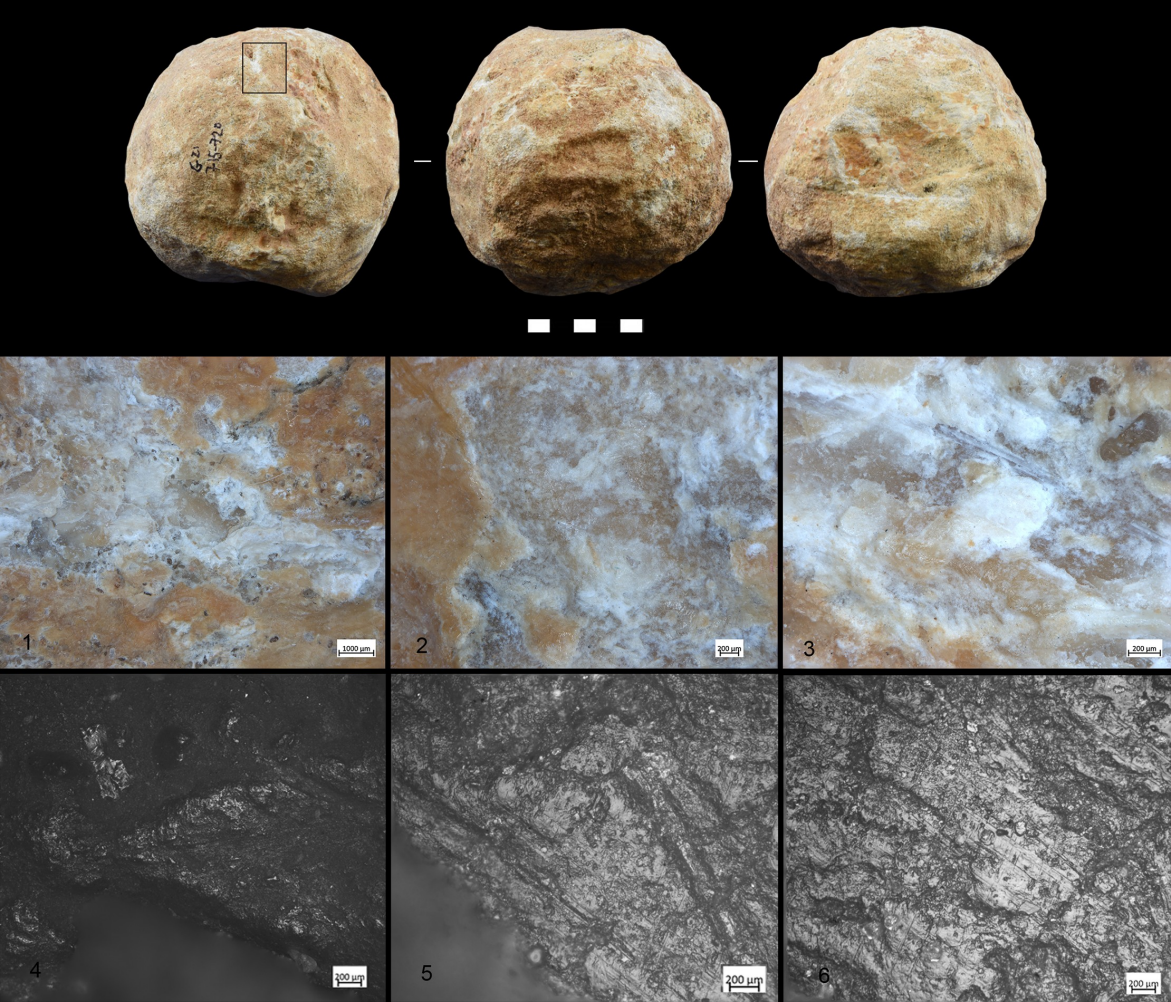
Archaeological SSB (QC G21 715–720). 1–3) Macro-traces characterized by oriented striations and negatives of flakes; 4) Localization of micro-polishes on the high ridges; 5 and 6) Abrasions and micro-striations with different orientation suggesting a repetitive gesture with different directions. The micro-traces documented in this figure were analysed on the silicon cast (Provil Novo®).

**Fig 7:**
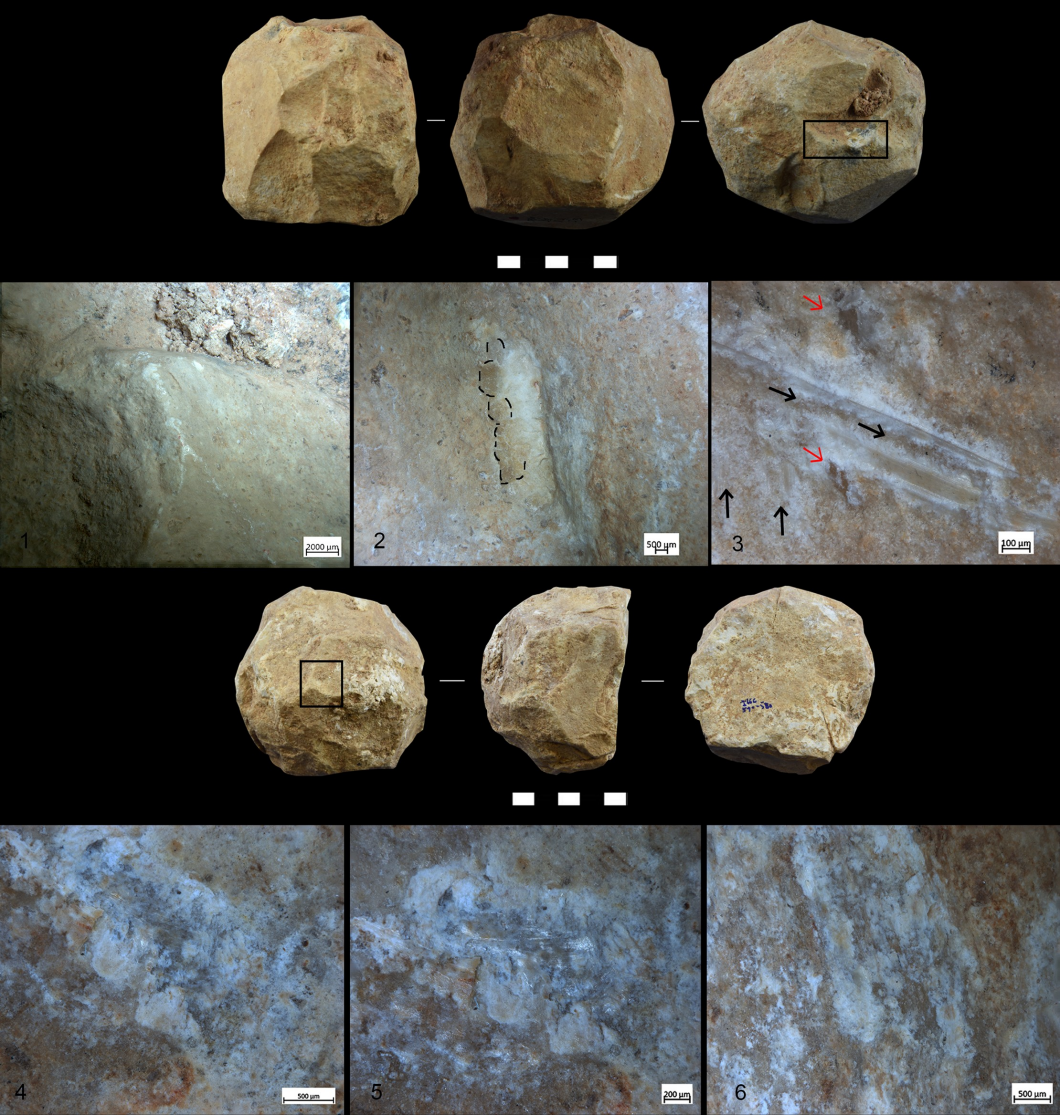
Archaeological SSB. QC H21 655–660:1) Macro-traces localised on high ridge; 2) Flake negatives; 3) Oriented striations (indicated by black arrows) with different directions and negatives of micro-flakes (indicated by the red arrows). Magnification 40x. **QC I16c 570–580**: 4–6) Cracks on the patinas due to the formation of new traces.

A variety of macro-and micro-wear traces were observed on the (technologically) shaped, angular ridges of the SSBs (Fig 5): Micro-flake detachments on nine items; long and deep macro-striations on four items; sheen/translucent appearance on six items; and leveled areas on two items (see Table 2 and Figs 4–7 for specific information on each tool). Micro-polishes were also localized on the top of the prominent ridges and characterized by features such as half-tight linkage, smooth texture, and domed or flat topography (Fig 5.4). Micro-striations were also observed. Overall, macro and micro-traces are consistent with characteristics expected from hard contact between the tools and fresh bone in thrusting percussion. Polishes with different orientations were also observed, suggesting a repeated activity and overlapping gestures, which led to the formation of abrasions on the micro-polishes characterized by rough texture (Figs 6 and 7).

**Table 2:** Archaeological sample data. Morphology, dimensions, raw material (calcareous rock in this case is either limestone or dolomite), use-wear and residues of the SSBs from Qesem Cave site.

ID	Type	Morphology	Raw material	L (mm)	W (mm)	T (mm)	W (g)	Use-wear		Residues
								Macro-traces	Micro-traces	
19. G21 670–675	Half-ball	Hexagonal/plane-convex section	Carbonate rock	80	79	51	447	Negative of micro-flakes localized on the high ridges; levelled area; sheen/translucent appearance	No silicon cast	Glossy and striated organic film. Spots of crushed amorphous white residues consistent with animal fat and fibers.
21. C9 890–900	SSB	Spheroid	Flint	84	82	87	902	Rounded high ridges	Polish with smooth/domed texture and topography	No residues
13. F22 735–740	SSB	Spheroid	Carbonate rock	85	93	87	1042	Rounded high ridge; macro-striations; negative of micro-flakes on the high ridges; oriented flat surfaces; sheen appearance	No silicon cast	Crushed compact and spongy bone tissues
18. H21 630–635	Half-ball	Subcircular/plane-convex section	Carbonate rock	67	80	72	632	Rounded high ridge; high ridges with negative of micro-flakes; macro-striations	No silicon cast	A few crushed compact and spongy bone tissues
17. H21 655–660	SSB	Hexagonal/ quadrangular section	Carbonate rock	72	78	78	629	Negative of micro-flakes related with the high ridges	No silicon cast	Crushed compact and spongy bone tissues. Spots of greasy fat animal matter mixed with bone fragments
28. E1	Half-ball	Circular/plane-convex section	Carbonate rock	94	97	46	514	Negative of micro-flakes; macro-striations; rounded high ridge: oriented flat surfaces; sheen appearance	No silicon cast	No residues
15. G21 715–720	SSB	Circular/oval section	Carbonate rock	82	81	86	818	Negative of micro-flakes; macro-striations	Smooth/flat micro-polishes appear on the high ridges. Abrasions on the oriented polishes are present, as result of overlapping gestures that removed evidence of the preceding traces. In this case, the polish shows a rough texture	Glossy and striated organic film. Spots of crushed amorphous white residues of animal fat and fibers
7. E17d 715–720	Half-ball	Hexagonal/ plane-convex section	Carbonate rock	78	78	46	360	Rounded high ridge; macro-striations; negative micro-flakes	Patches of micro-polishes with smooth texture and flat topography and striations, with the same orientation	Glossy and striated organic film. Spots of crushed amorphous white residues of fat and fibers. Crushed compact and spongy bone tissues. Spots of greasy fat animal matter mixed with bone fragments
26. I16C 570–580	Half-ball	Hexagonal/ plane-convex section	Carbonate rock	65	68	49	318	Negative of micro-flakes; levelled area; rounded high ridges	No silicon cast	Glossy and striated organic film. Spots of crushed amorphous white residues of animal fat and fibers. Crushed compact and spongy bone tissues. Spots of greasy fat matter mixed with bone fragments
8. F15C 715–720	Quadrangular pebble	Sub- rectangular/ plane-convex section	Carbonate rock	79	90	53	612	Negative micro-flakes; rounded high ridges	Micro-polishes appear as smooth texture and domed topography, localized on the top of high ridges, associated with long micro-striations	Glossy and striated organic film. Spots of crushed amorphous white residues of fat and fibers. Crushed compact and spongy bone tissues. Spots of greasy fat matter mixed with bone fragments

Residues were exceptionally preserved on eight tools and distributed across their surfaces. Archaeological residues have morphological features, appearance, color, and distribution compatible with compact and spongy bone, organic bone glossy film, collagen fibers, and animal fatty matters observed on experimental stone balls used in bone marrow extraction activities. In particular, on the top of the prominent ridges, residues appear as spots of organic film with a glossy and often striated appearance. They coexist with spots of crushed amorphous white residues consistent with bone fat and collagen fibers (Fig 5A and 5B) sometimes also smeared and crushed onto the tools’ scars. Here, residues appear as particles of compact and spongy bony tissues associated with a greasier substance (Fig 5C–5E). Additionally, patches of crushed greasy matter mixed with compact bone fragments, displaced and reaggregated during the activity, were identified on the flat surfaces of two SSBs (Fig 5F).

### Experimental framework

A preliminary evaluation of the nature of use-wear and residue on the archaeological SSBs has been conducted using percussion tools from the experimental reference collection at DANTE—Diet and Ancient Technology Laboratory in Rome (Sapienza University). Overall, this preliminary experimentation allowed us to evaluate patterns of use-wear and residue distribution while attempting a preliminary functional evaluation of the material worked using the SSBs from Qesem cave. The replicas of percussion tools had prominent ridges and morphology similar to the archaeological finds and were used to process different types of organic matter.

In addition to the abovementioned reference collection, two more experimental trials were conducted in order to investigate and test specific functional features of the archaeological finds.

The first experimental trial (EXP-1) was conducted by IC, CL, AG and EA as a way to test the efficiency of limestone unshaped cobbles compared to shaped cobbles in processing fresh bones by thrusting percussion. Results of this trial were key role for interpreting macro and micro-traces observed on the archaeological SSBs. The experimental activities were chosen on the basis of the archaeological evidence, namely the use-wear and residues observed on the Qesem SSBs as well as considering the rich assemblage of animal bone recovered at the site [41–42]. Three limestone cobbles collected at Wadi Qana (5 kilometers north of the cave) were used in this experiment (Table 3). The experiment focused on hammerstone percussion, which is produced when the bone rests on the ground and is hit with a stone ball.

**Table 3:** Unmodified cobbles used in EXP– 1.

Unmodified limestone cobble	Length (mm)	Width (mm)	Thickness (mm)	Weight (g)
1	120	91	30	810
2	114	67	80	875
3	93	82	70	805

The second experimental trial (EXP-2) was conducted by JR, RB, IC, AG and EA as a way to specifically test the efficiency of SSBs in fresh bone crushing activity aimed at extracting bone marrow. Through this trial, macro-detachments developed on SSBs, sometimes changing their morphology, were documented together with diagnostic use-wear features and residues produced during the specific activity.

**Fig 8:**
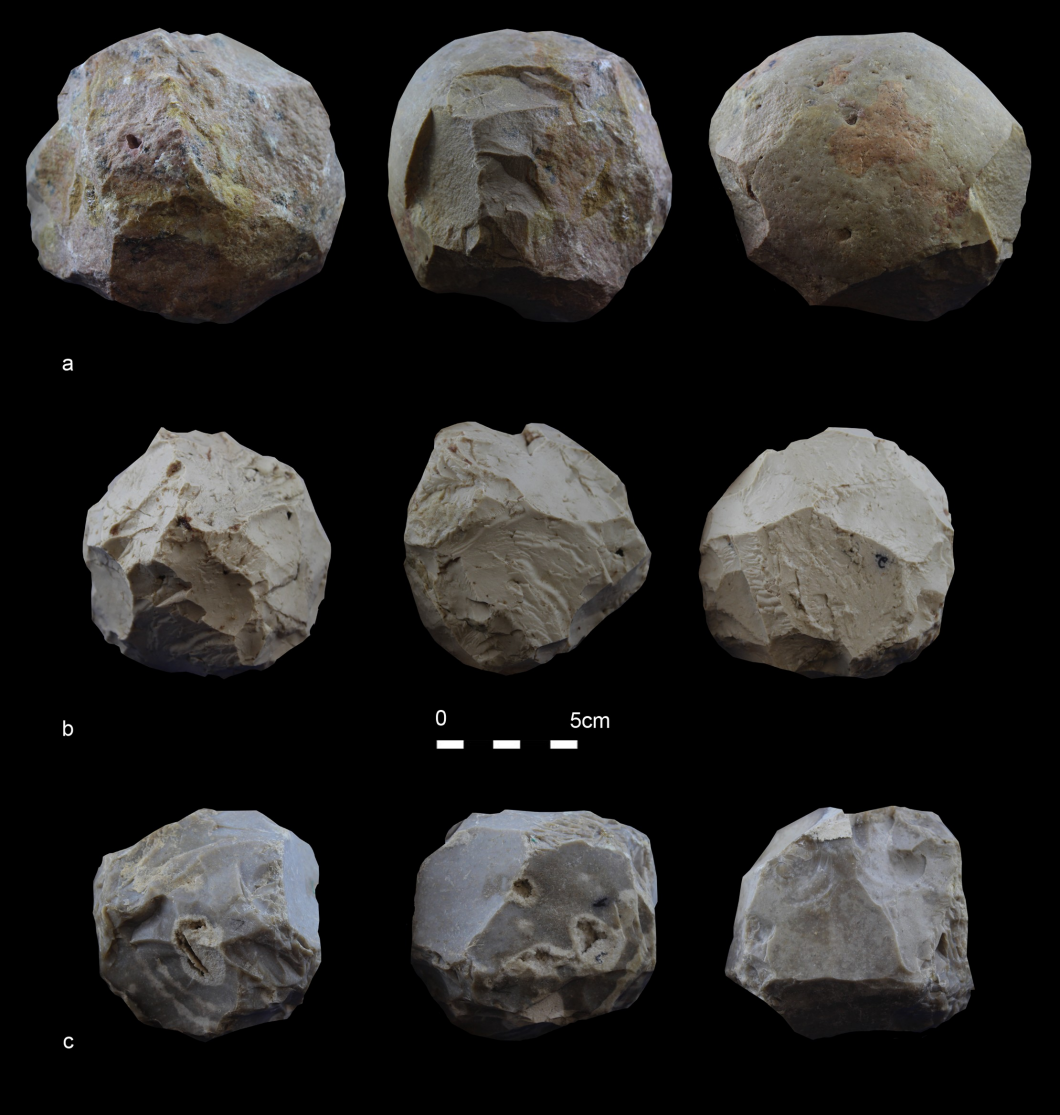
Experimental SSB replicas. The replicas were knapped by J. Baena and employed in marrow extraction. a) Large size dolomite SSB; b) Medium-size limestone SSB; c) Medium-size flint SSB.

Three replicas were produced by JB and used in this experiment (Table 4, Fig 8) together with one natural limestone cobble collected at Wadi Qana.

**Table 4:** Items used in EXP– 2.

Replica	Type of material	Source of material	Length (mm)	Width (mm)	Thickness (mm)	Weight (g)
1. Large size	Dolomite	Wadi Qana, Israel	140	160	135	2237
2. Medium size	Limestone	Miocene east area of outcrops south of Madrid	90	77	85	806
3. Medium size	Flint	Miocene east area of outcrops south of Madrid	90	69	76	730
4. Natural cobble	Limestone	Wadi Qana	114	67	80	875

In the case of replica n.2 carbonate rock selected was not retrieved from the immediate surroundings of Qesem Cave. As previously stated, a preliminary geological survey indicated that the limestone originating from the natural formation of the cave itself (the Turonian Bi’na Formation [77]) was of low-quality, weathered and abraded. While reconstructing the knapping sequence for producing SSBs goes beyond the scope of this article, the series of technical operations involved in their experimental manufacturing (e.g. the creation of a flat, right-angled surface and, ultimately, of a symmetrical, spherical morphology) would have certainly required good-quality raw materials. As local stones broke during the experiment, durable limestone cobbles with similar performing characteristics to the archaeological samples were collected by JB in the Miocene east area of outcrops south of Madrid and used to create the rest of the experimental tools. These limestone cobbles originate from Jarama-Tajuña-Tajo rivers basin and they belong to the Upper Unit of the neogenic landfill of the Miocene Madrid Basin [78]. In the future we intend to produce few more SSBs from materials in the vicinity of Qesem Cave and conduct further experiments.

During the EXP-2 different variables were taken into consideration such as the tool size and material, the size of the bone, and the anatomical part chosen for the experimentation. In order to test whether skill levels influenced the effectiveness of SSBs in general, two experienced and three unexperienced individuals participated in the experimental session of bone breaking.

**Fig 9:**
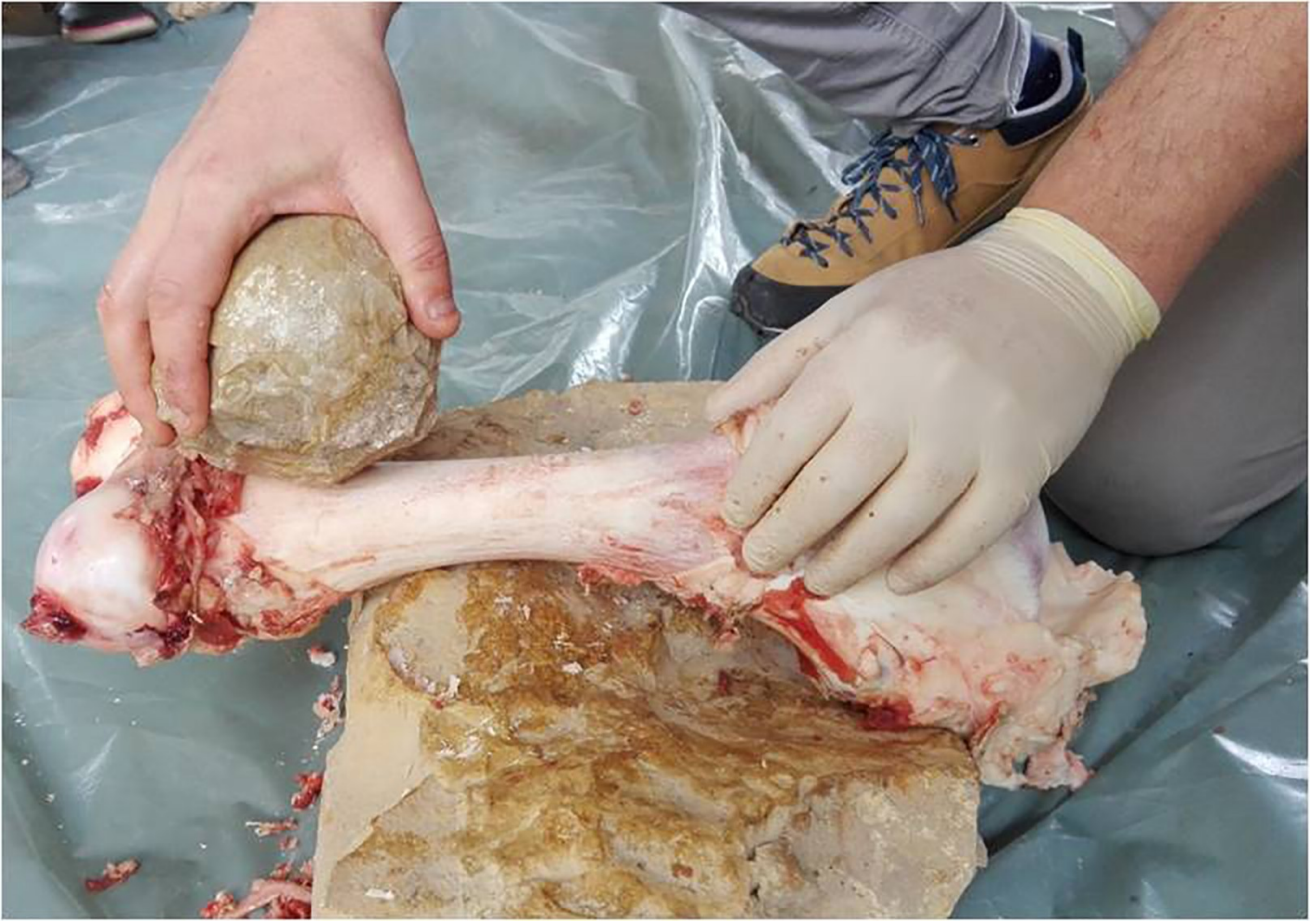
Marrow extraction experiment. A large dolomite SSB used for bone breaking in order to extract the marrow (performed by J. Rosell).

**Fig 10:**
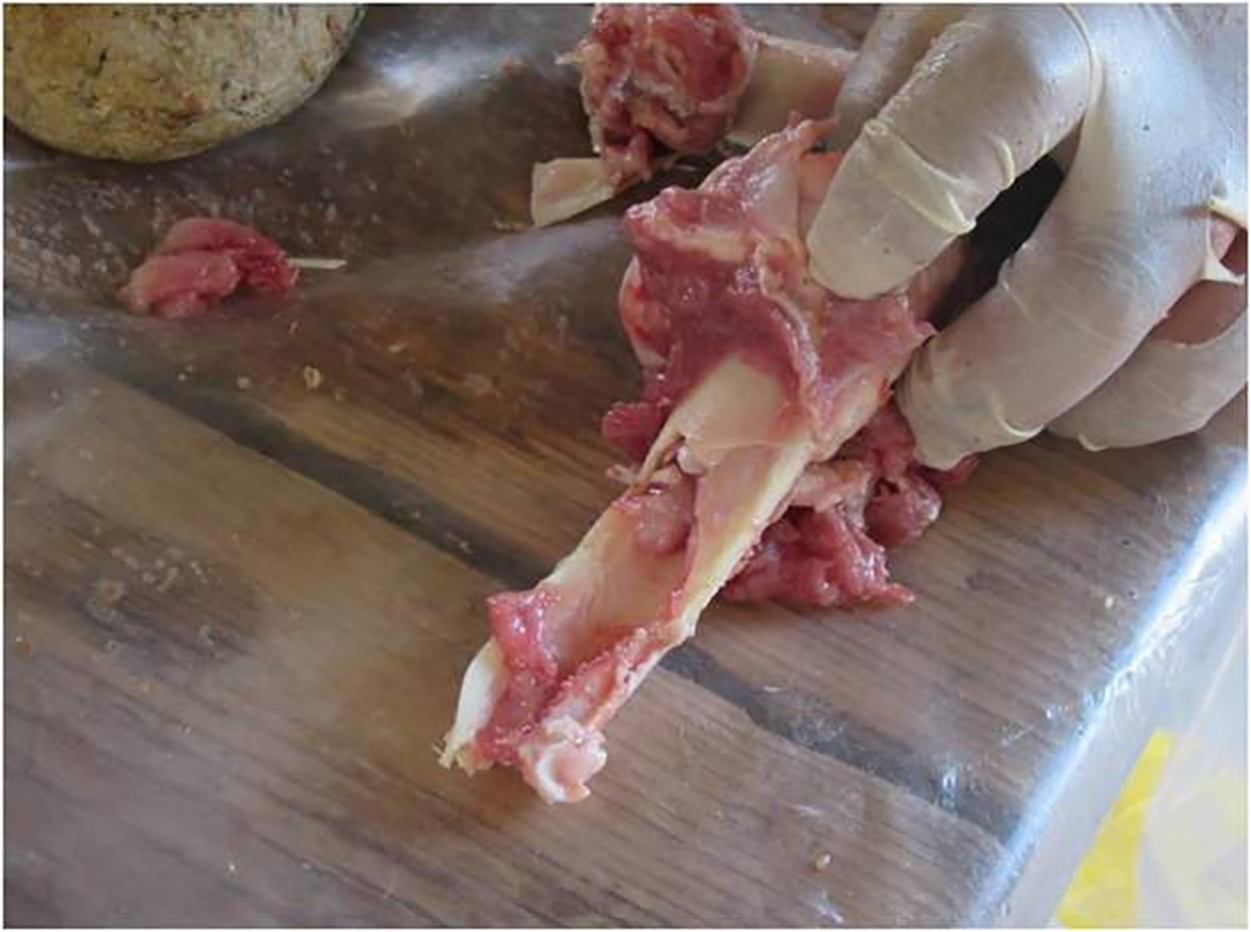
Bone marrow extracted with an experimental SSB.

All bones were prepared by removing the periosteum using a flint flake. A total of 11 bones were broken (4 cow femora and 5 cow humeri, 1 sheep humerus and 1 sheep radius-ulna). All individuals broke the bones by means of direct hammerstone percussion, including the use of four SSB replicas (Fig 8) with the objective of extracting the bone marrow under optimal conditions. An anvil of 38cm length, 23–26 cm width, and 16–20 cm thickness was used to stabilize the bone prior to hitting (Fig 9). During the test, the macro-tools were mainly used for crushing fresh bone using a repeated gesture of thrusting percussion. Experimenters focused on the use of the prominent ridges of the SSBs, and through this gesture marrow extraction was facilitated (Figs 9 and 10). The time per bone breakage and the number of the experimental tool as well as the experimenter were systematically recorded; notes related to each experimenter were taken using photos and videos.

#### Use-wear and residue analysis on bone-crushing experimental sample

The SSB replicas were found to be very efficient for crushing animal bones (mainly due to the prominent ridges, which are non-existent in the natural cobbles), and no impact led to a significant change of their original morphology.

**Fig 11:**
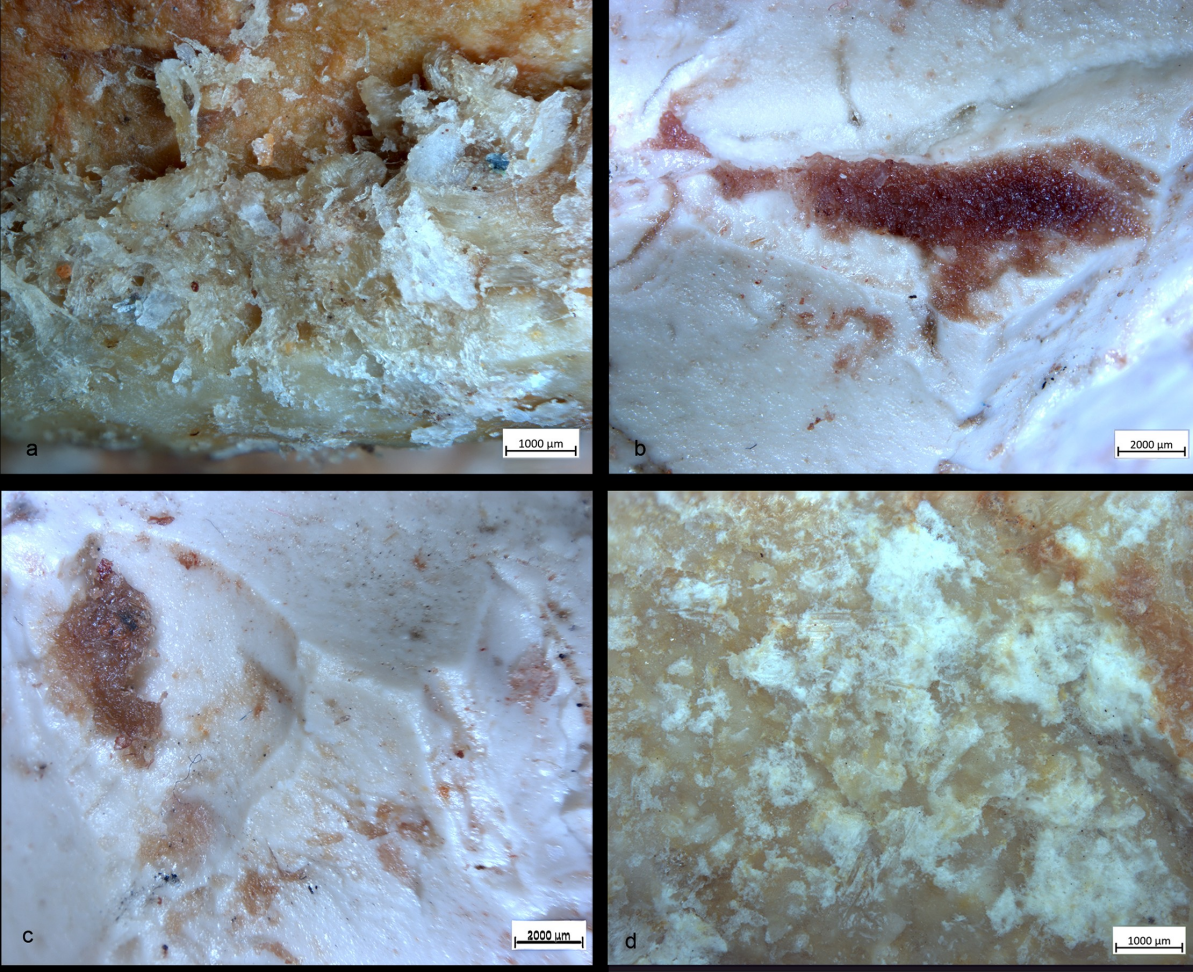
Experimental residues related to bone crushing. (a) Collagen fibers and fragments of periosteum tissue localized on the top of the high ridges; (b,c) Amorphous reddish (meat- and blood-rich) animal matter compressed inside the negative scars; (d) close-up on amorphous whitish animal matter. Note the formation of an organic film with a glossy and striated appearance on top of the residue.

Residues associated to bone crushing were localized on the prominent ridges of the experimental replicas as well as inside the negative scars related to the technological procedure of tool shaping. Four different types of residues have been recognized: (a) collagen fibers and fragments of periosteum tissue; (b) amorphous reddish animal matter; (c) amorphous whitish animal matter; (d) small particles of crushed bone. Collagen fibrils were abundant and often found in association with particle of periosteum tissue (Fig 11). Their distribution was mainly confined to the top of the ridges while their compressed and/or smeared appearance was certainly indicative of the repetitive percussion gestures applied by the experimenter. Amorphous reddish matter mainly composed of meat and blood was abundant and widely distributed all over the surfaces of the experimental tools. While the specific round shape of the SSBs certainly affected the way tools were manipulated and, consequently, the wide distribution of softer residues across the whole tools’ surface, patches of reddish meat-rich amorphous matter adhered particularly to the flatter and more concave areas of the SSBs. In particular, the reddish amorphous material appeared packed and compressed inside the negative scars related to the tool shaping (Fig 11B). In addition to this, whitish amorphous bone- and fat- rich matter was also distributed on top of the ridges, appearing either compressed or smeared with clear directionality on the flatter surfaces (Fig 11D). The friction between the experimental SSB stone surface on the bone also produced a very specific tribological alteration of such whitish amorphous material, which acquired in some cases a glossy-like appearance (Fig 15A). A similar modification has already been identified and associated to the heat generated on the residue during bone processing [79]). On our experimental record, the correlation between the formation of a glossy film on top of the residue and use-related traces is suggested by the co-presence of compressed spots of glossy matter deeply striated due to the friction (Fig 11D). A similar modification possibly produced by the interaction between residue and specific use-traces (striations) has positively been identified on the archaeological SSBs analyzed in this article (e.g. Fig 11B). Last, small particles of crushed bones were also sporadically identified amongst the experimental residues. Such type of residues was identified on flat surfaces as well as inside the negative scars related to the tool shaping in association with compressed amorphous fat-rich as well as compressed collagen fibers (Fig 15C).

**Fig 12:**
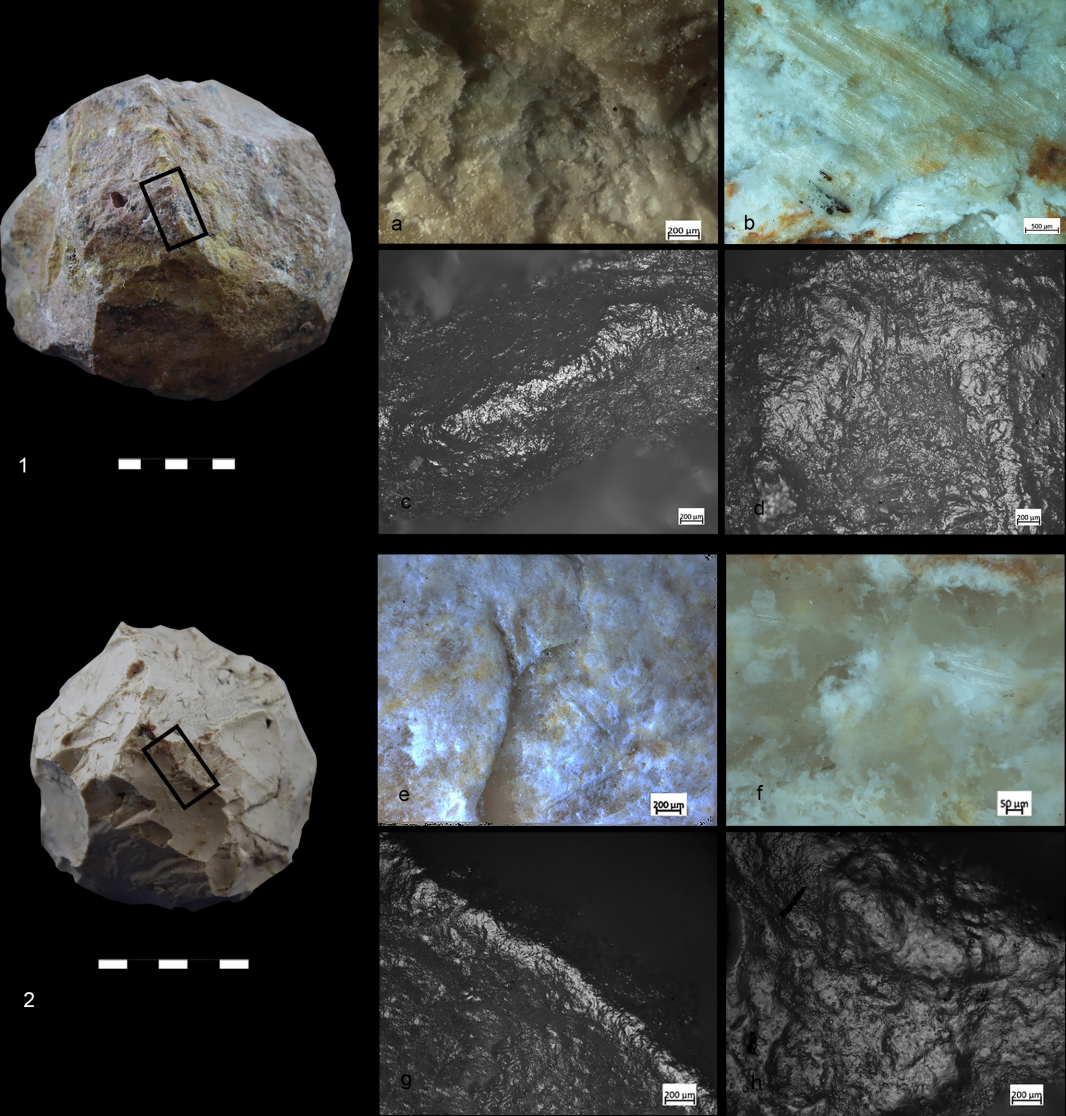
Experiments used for crushing bone. 1) Limestone spheroid with macro- and micro-wear: b) Medium-size spheroid in compact limestone related to the macro and micro traces.

**Fig 13:**
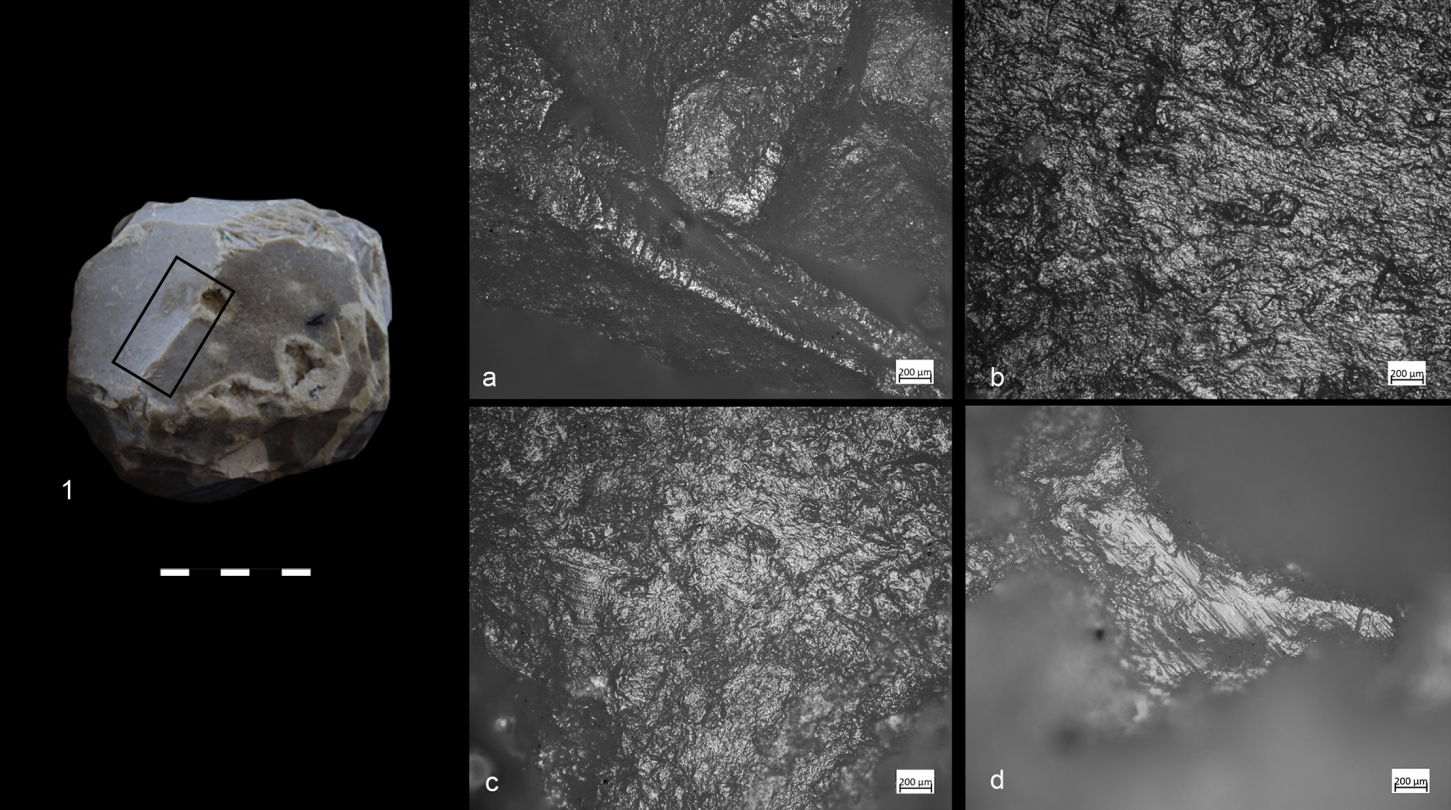
Experiment used for crushing bone. Flint spheroid and characteristic micro-traces localized on the high ridges associated with striations.

Macro-traces were localized on the prominent ridges of the tools (Fig 12), which appeared as rounded at 10x of magnification. At 40x–50x of magnification it was possible to observe negatives of micro flakes and levelled areas. The micro-polishes were localized on the top of the grains, with half-tight linkage, smooth texture, and domed topography (Fig 12). In some cases, micro-striations characterized by a similar orientation as the one characterizing the use-wear overlapped the patches of polish. Abrasion affected the polish resulting in a rough topography (Fig 13).

Interestingly, no macro-flakes such as those observed on the archaeological sample developed on the experimental replicas (at 20x of magnification). Different explanations could explain the discrepancies with the archaeological data. In particular, experimental results could be due to the specific force and types of gestures used by the experimenter during the percussion activity; to the size and hardness of the crushed bones; and, finally, also to the repetitiveness of the actions performed. Understanding entirely the formation of these variables goes beyond the scopes of this article and would require a wider and more detailed experimentation.

## Discussion and conclusions

**Fig 14:**
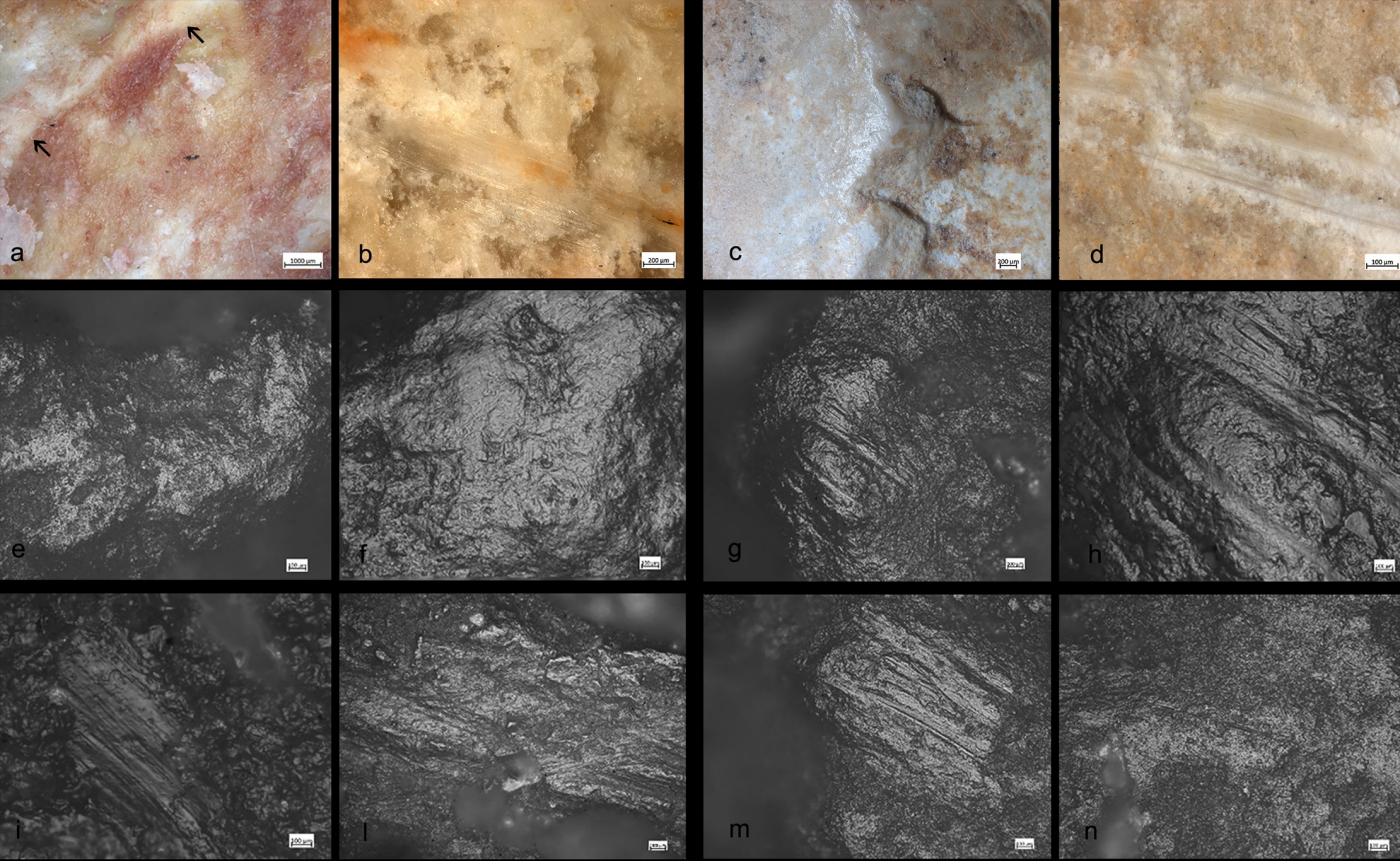
Comparison between experimental (left) and archaeological (right) use-wear. *Experimental traces (a*,*b*,*e*,*f*,*i*,*l)*: The black arrow indicated use-wear developed on the top of the high ridges (a); Small-flake detachment associated with oriented and striated residues characterized by a glossy-like appearance (b); Micro-polish localized on the high ridge (e) with smooth texture and domed topography (f); Patch of polish with micro-striations with the same orientation (i); Overlapping polishes with different orientation and rough aspect (l). *Archaeological traces* (c,d,g,h,m,n), localization of the use-wear on the high ridge (c); Oriented residues and striations (d); Micro-polish localized on the high ridge (g) with smooth texture and domed topography (h); Patch of polish with micro-striations with the same orientation (m); Overlapping polishes with different orientation and rough aspect (n).

Use-wear and bone residues on ten SSBs indicate that the inhabitants of Qesem Cave favored the use of shaped, somewhat angular, stone balls made of carbonate rocks to crush fresh animal bones to access fat: mostly marrow and possibly grease too (Figs 5, 7, 12 and 14). Patinated and even broken SSBs were selected from outside the cave and brought in for this specific activity. These tools still exhibit some of their unique characteristics, such as semi-rounded morphology and ridges. Our experiments showed that SSBs are indeed efficient for bone processing, providing a comfortable grip and useful active areas with several suitable working edges for repeated use. In particular, their morphology and the way they were manipulated (e.g., rotated during use) seem to have affected the use-wear and residue distribution across the surface of the tool. For the first time, our analysis and experimental data support a link between the functional and morphological traits of these intriguing Paleolithic items.

**Fig 15:**
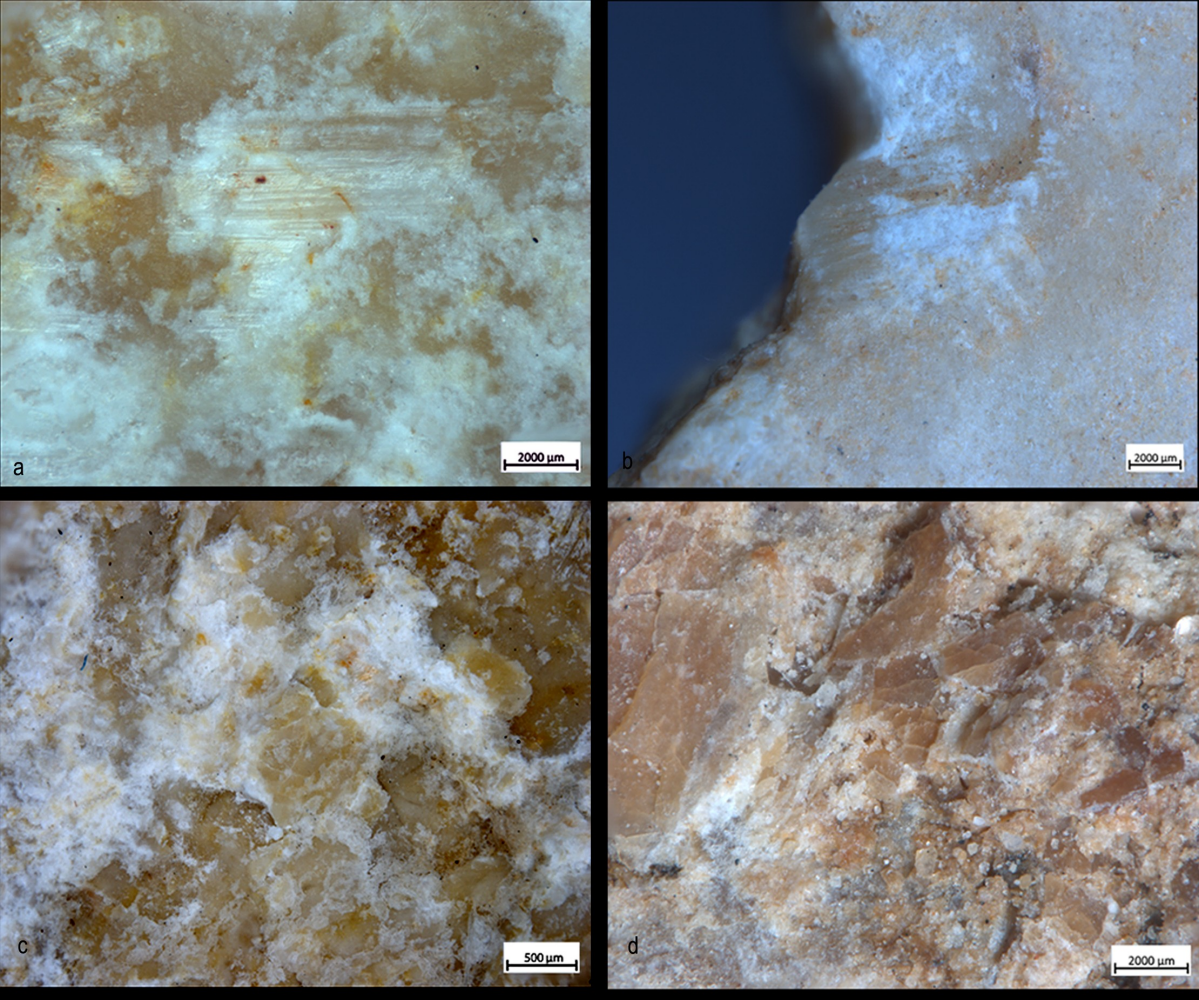
Comparison between experimental (left) and archaeological (right) residues. *Experimental residues (a*,*c)*: close-up on amorphous whitish animal matter and the organic film with a glossy and striated appearance on top of the residue (a); White amorphous material composed of fat compressed and admixed with small particles of crushed bone (c). *Archaeological residues (b*,*d)*: Compressed whitish amorphous material with organic glossy and striated film formed on top (b); Crushed bone compact tissue mixed to small amount of amorphous whitish residue with a compressed aspect (d).

In particular the experimental results indicate that the level of experience in bone breaking certainly affected the time required for processing the bone. We thus infer that planning, precision, and know-how were required for selecting adequate tools and for properly using them for the task. Remarkably, damage related to bone breakage activities is observed on the faunal record throughout the stratigraphic sequence of the cave, while SSBs were found only in particular Amudian contexts. At this stage of our research, it is still early to provide a coherent explanation for this state of affairs. However, we do not argue that bone breakage at the cave was accomplished only by the use of SSBs, and it is most reasonable that other hammerstones were used for this purpose as well (e.g. pounding implements, as discussed in the work of de Beaune [8] and de la Torre [80]). Nonetheless, as SSBs seem to be long-lasting, efficient bone-breakers, it could well be the case that these items were used in different contexts inside the cave and then discarded at specific (designated) locations (these issues and research questions are on the agenda and will be further investigated).

To date, use-wear traces and organic residues observed on ten SSBs from Qesem Cave are the only (and earliest) direct evidence of the use of this type of artifacts. They clearly confirm the function of SSBs and their role within the toolkit of Qesem Cave inhabitants. The people of Qesem were well-acquainted with the landscape around the cave, which was also favored by earlier Lower Paleolithic groups. Thus, the landscape was rich in relics of earlier groups, some of which were well-suited to the needs of the cave’s inhabitants. These patinated relics might have been selected from ancient Acheulian sites, e.g., the late Acheulian sites of Jaljulia or Eyal, located 6–12 km north of Qesem (see [81]), the late Acheulian site of Revadim quarry, located 40 km south of Qesem, in which several SSBs were also found, or other still unknown sites in the region. The faunal record of Qesem Cave indicates a continuous fat-oriented use of prey for dietary purposes [82]. Fat is thought to have been a significant component of foragers’ diet [6, 83], and specifically marrow provides the greatest percentage of fatty acids within the whole animal body [83–84]. It was therefore preferred by early humans throughout the Lower Paleolithic. Our study highlights the significant role SSBs played in this arrangement and underlines the importance the residents of Qesem Cave attributed to the extraction of bone marrow. As efficient implements for fat extraction, these items might have helped enhance human caloric intake and adaptation in the Lower Paleolithic period.

## Supporting information

S1 Data (DOC)
