# Supplementary material for: Shaped stone balls were used for bone marrow extraction at Lower Paleolithic Qesem Cave, Israel
Source: PLoS One. 2020 Apr 9;15(4):e0230972. doi: 10.1371/journal.pone.0230972 (PMC7145020; doi:10.1371/journal.pone.0230972)
Supplement: S1 Data — (DOC) [file pone.0230972.s001.doc]

**This file includes:**

- The SSB sample: detailed archaeological contexts (Figs S1-10).
- Detailed sample description.
- Analysis of faunal results produced through the experimental trials (Tables S1-2).
- References (RS-17).

**The SSB sample: detailed archaeological contexts**

Twenty-nine SSBs were found at Qesem Cave. The stratigraphic sequence of the cave is generally divided into two parts: lower (∼7 m thick), consisting of sediments with clastic content and gravel, and upper (∼4.5 m thick) mostly consisting of cemented sediment with a large ashy component. The lower part was deposited in a time when the cave was a closed karstic environment, while the upper part was deposited when the cave was more open as indicated by the presence of calcified rootlets [RS1]. The SSBs were concentrated in particular, mostly Amudian contexts of the lower stratigraphic sequence of the cave [RS2].


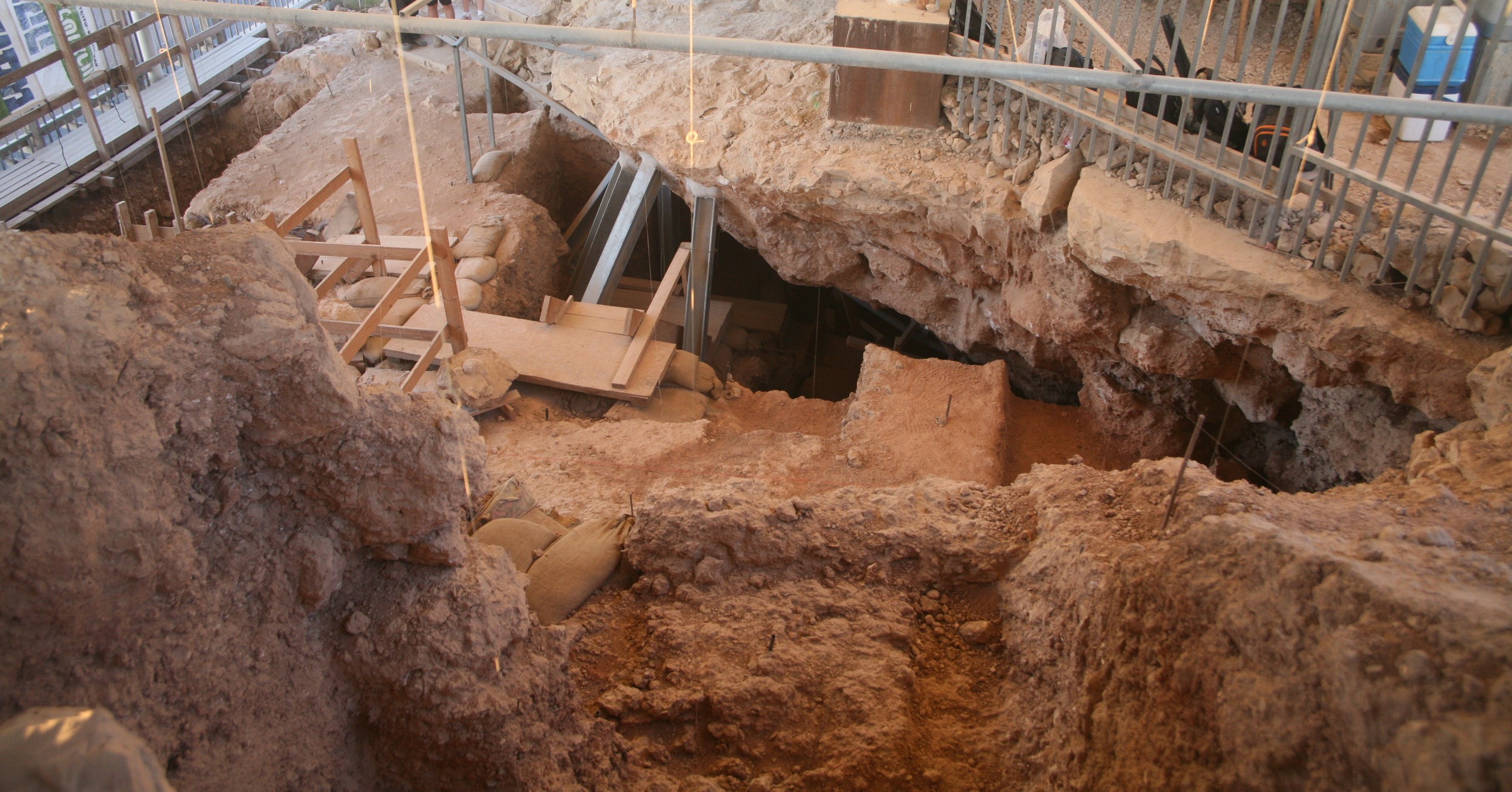


**Fig S1.** General view of Qesem Cave, a view from east to west, excavation season 2012.

One group, comprising nine items, was discovered in the southern part of the cave (in the lower sequence) in an area restricted to about five square meters, between elevations of 625-740cm below datum. Two items were found within a single square meter at a very similar elevation. The items from this area originate from squares F/22, G/20 G/21, H/20 – these Amudian assemblages were excavated in an area that was damaged by the newly built road. One item was retrieved from the Amudian context of excavation square I/16 (which was not damaged by the construction). The layers in this area are located close to bedrock and are earlier than 300ka.


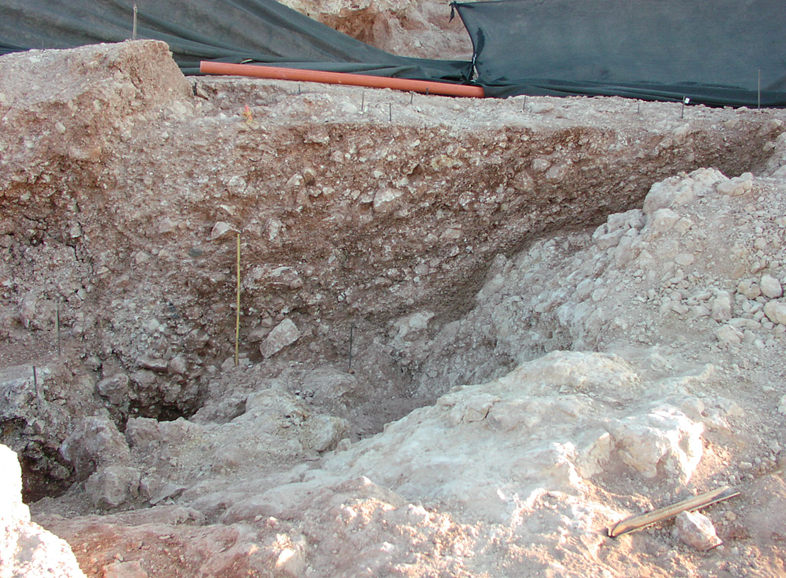


**Fig. S2.** **The southern areas of the cave (which was excavated during the salvage, first excavation season).** The SSBs were found close to the section and adjacent to the bedrock exposed in the southern part, where the road now passes.


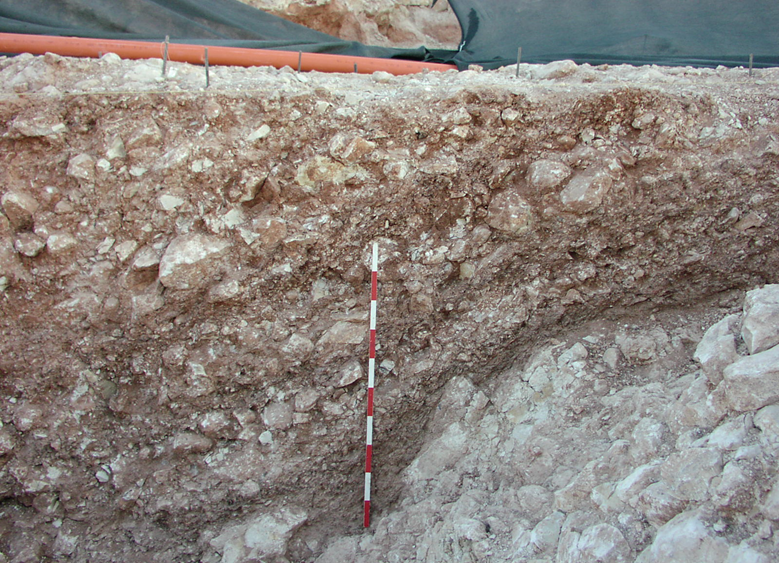


**Fig S3.** **A closer look to the southern area section.**

Another group, comprising ten items, was discovered in the south western area of the cave (an area of four square meters in the lower sequence of the cave) between elevations of 660-720cm below datum: four stone balls were found within a single square meter, two additional items were found within another one-half square meter, and two items were found within a third one-half square meter, all at similar elevations. These items were retrieved from Amudian assemblages that are older than 300ka.


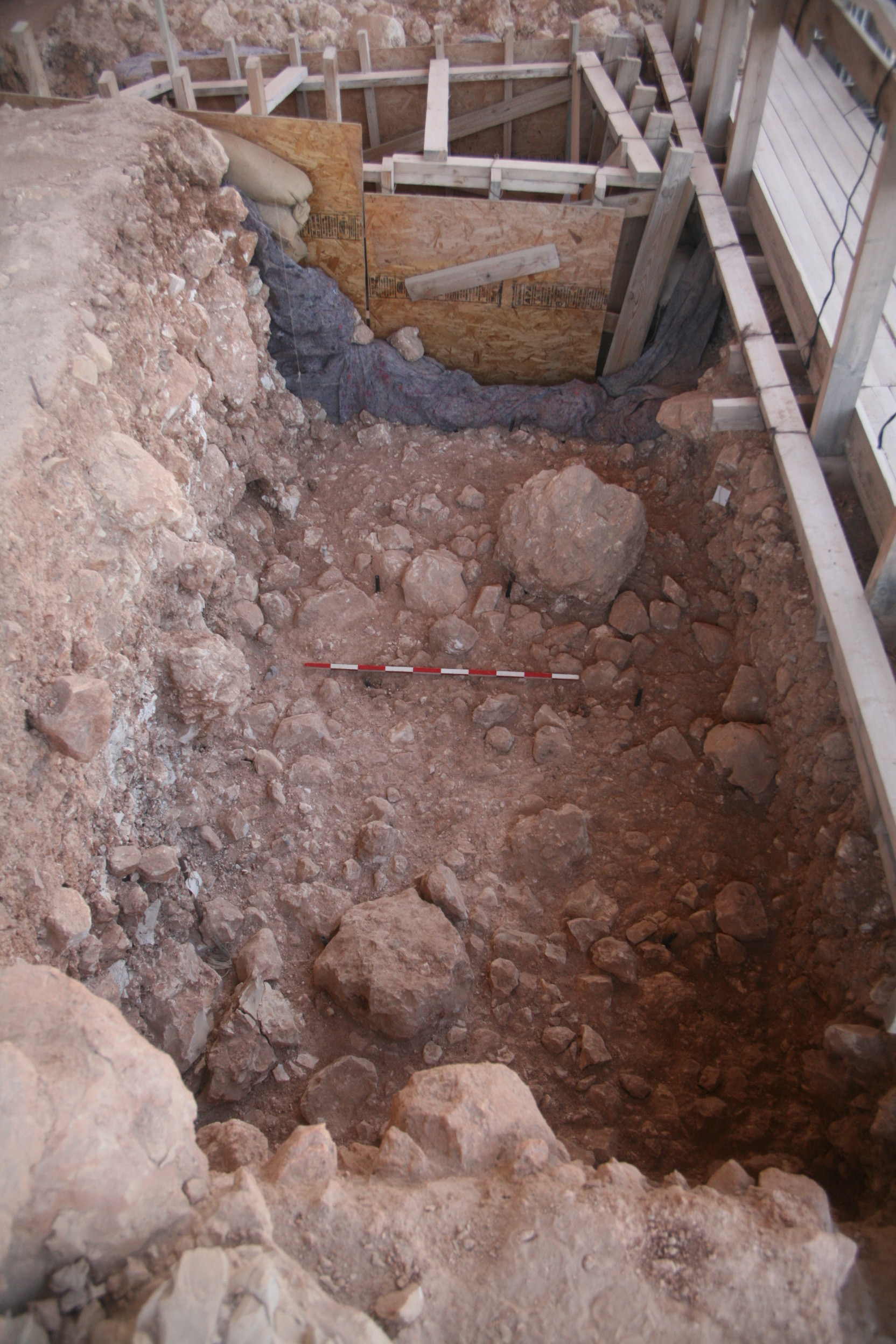


**Fig. S4.** **The south west area of Qesem Cave (a view from west to east, excavation season of 2014).**


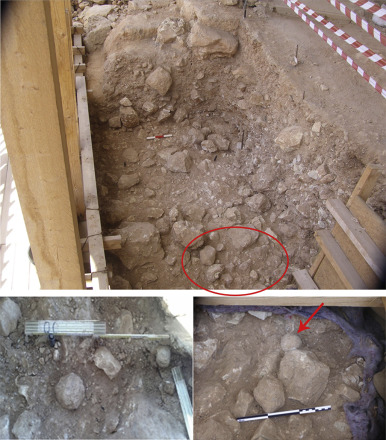


**Fig. S5.** **The south west area of Qesem Cave (a view from east to west, excavation season of 2014).** Ten items were found in this area (two items are marked with red arrows).

Two additional stone balls were found in a one-half square meter area adjacent to the central hearth from the south (in similar elevation on the stratigraphic sequence). The items were found between the elevations of 570-590cm below datum. This Amudian assemblage from the lower sequence of the cave is estimated as dating to 300ka [RS3].


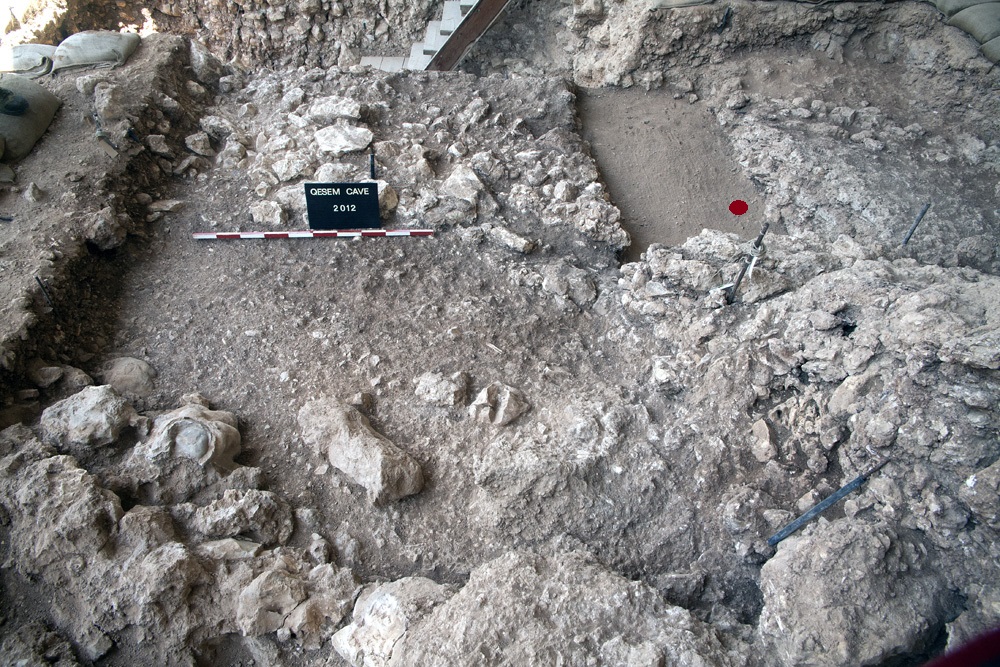


**Fig S6.** **The central hearth (marked by a red circle) and the area adjacent to it to the south (a view from east to west, excavation season of 2012).**

Six items were found under the rock shelf area in various excavation units and at different elevations.


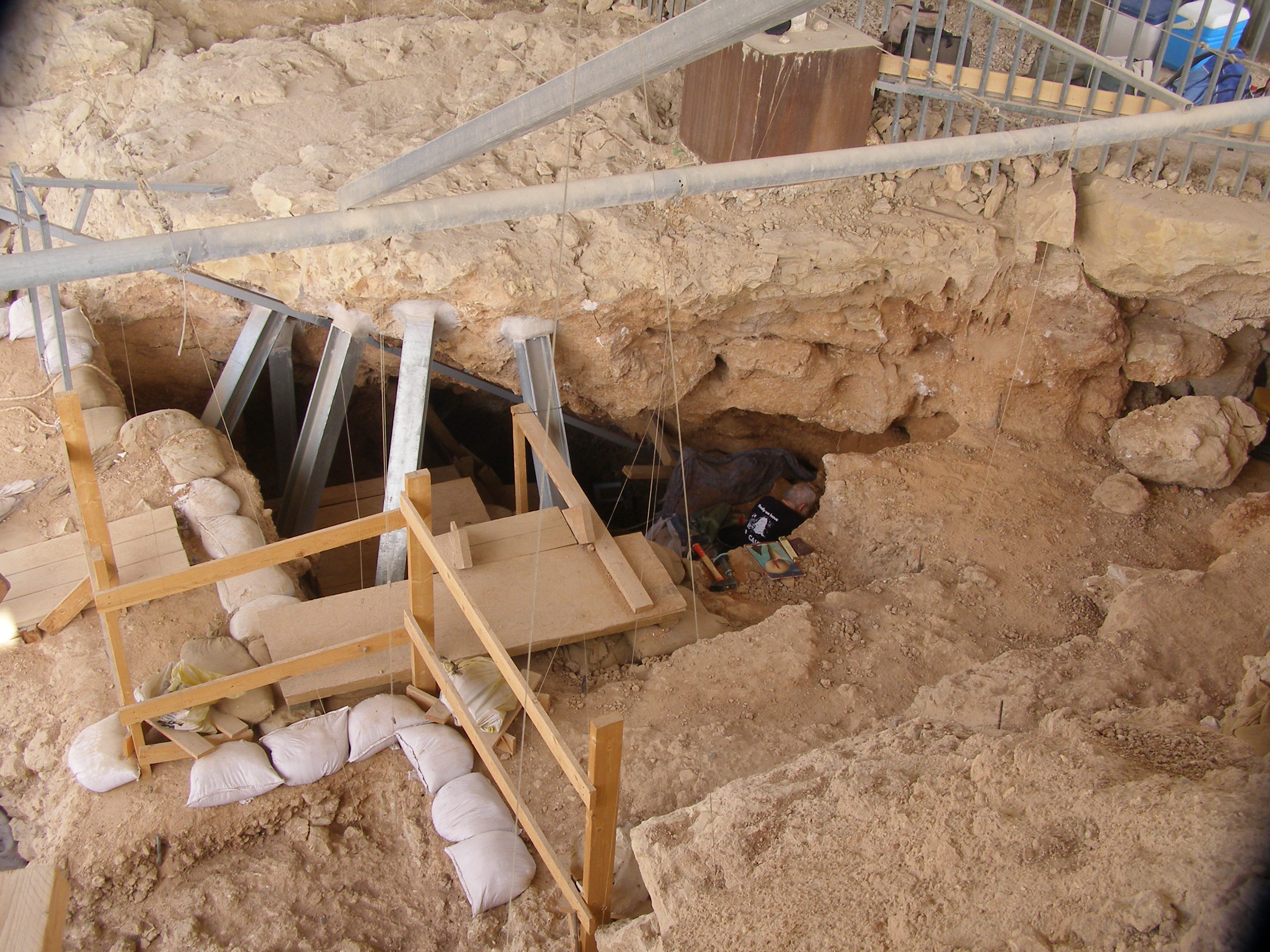


**Fig S7.** **The rock shelf (a view from east to west).**

The rock shelf consists of several distinct layers – the upper part of its sedimentary sequence was divided into two layers according to the sediment color and texture- both Yabrudian in composition [RS3]. Beneath, the sediments were divided into two stratigraphic layers, each containing a distinctive lithic industry - Yabrudian and Amudian [RS4]. One SSB was found in this Yabrudian context (G/9c 680-685, fig.1).


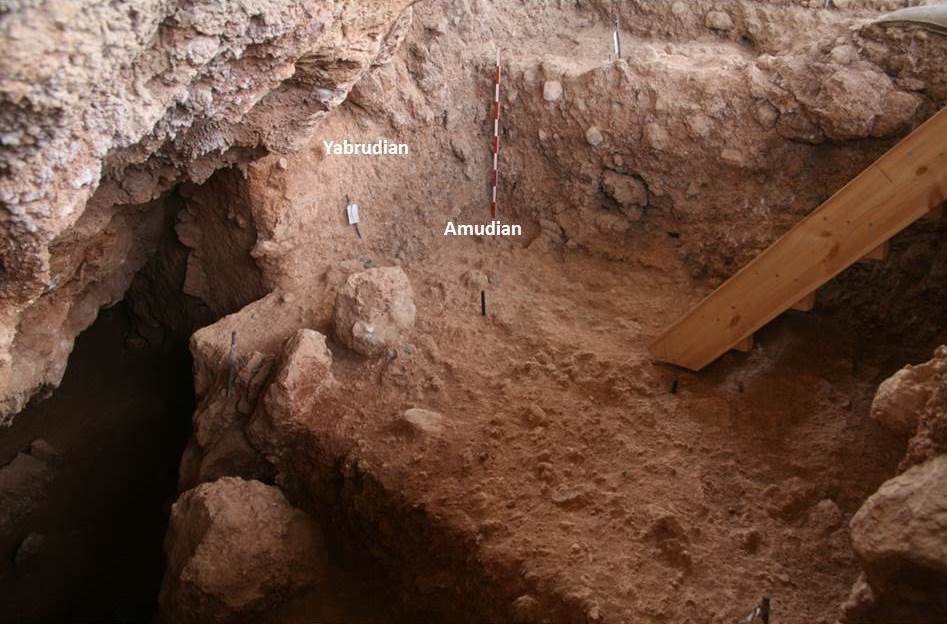


**Fig. S8**. **The upper Yabrudian and the Amudian layer beneath it, under the rock shelf (excavation season of 2010).**

Another SSB was found in an Amudian layer which lies beneath the Amudian context mentioned above (G/8b 700).


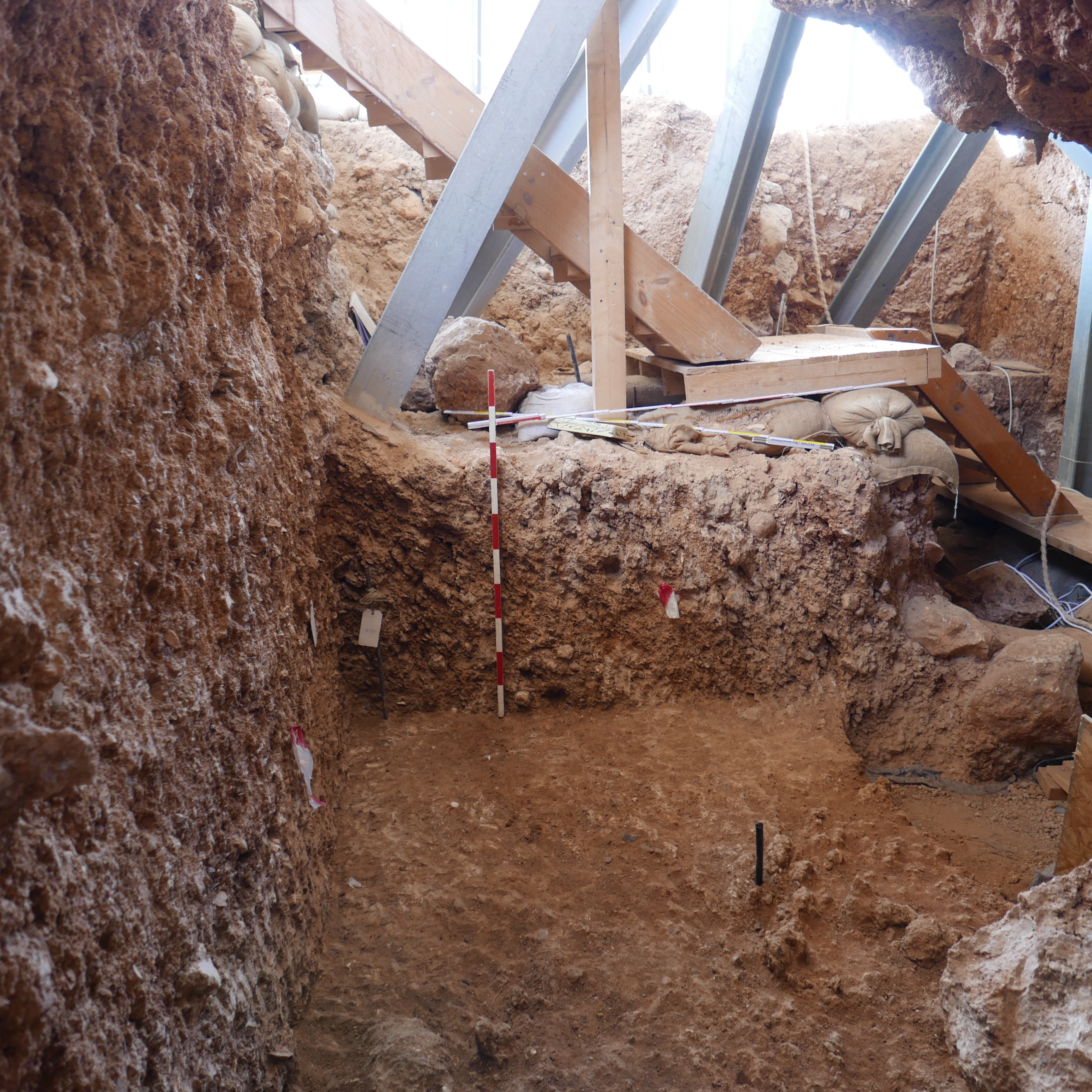


**Fig S9.** **The Amudian layer in the middle of the rock shelf (excavation season of 2016).**

Two items were found close to the eastern section of the rock shelf in Amudian contexts (C/7a and C/8 715-720). One item was found isolated with no association to other findings (C/9 890-900), and one item (D/7a 1130-1135) was found in the deepest assemblage of the cave, which was identified as Yabrudian but shows high frequency of laminar items ("Deep shelf unit 1").


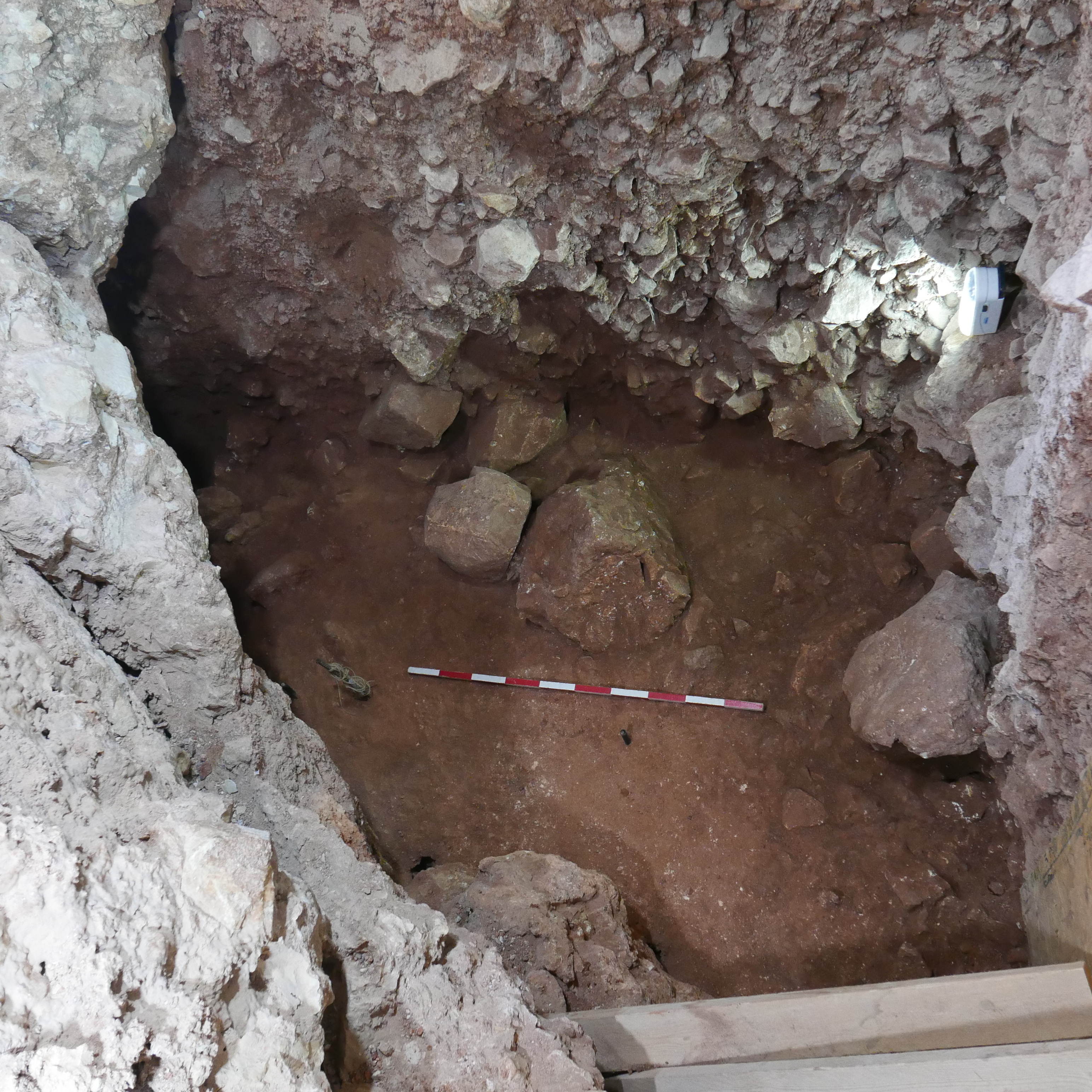


**Fig S10**. **The deep shelf unit 1 area – the deepest excavated area in the cave (excavation season of 2016).**

Two stone balls were collected from inside the cave within non-excavated contexts when the cave was first discovered. Qesem Cave was discovered during the construction of a road in the year 2000. Up until that moment it was sealed (at around 200,000 years ago – all layers were assigned to the Acheulo-Yabrudian Cultural Complex). Two SSBs included in this study were found in the course of the first salvage excavation at the site, on the surface of the archaeological layers inside the cave, following the removal of the ceiling of the cave by an explosion during preparatory work for road construction. These items were found inside the cave, and are part of the archaeological deposits of the cave, however due to the nature of the discovery of the cave and the destruction of the ceiling these items were not assigned to any specific archaeological context. Even though these items were not found in a well-defined, excavated context, they originate from inside the cave (which was sealed and undisturbed up until then). The fact that these items bear use-wear signs and residues attests to their good preservation state and therefore they were included in this study.

The type of calcareous rock from which the ten SSBs are made is not detectable with the naked eye, but requires a destructive action that will not be conducted at this point until the functional analyses are finalized. According to the geological observations (performed by O.B and E.Z) these items are made of limestone or dolomite, and one item is made of flint.

**Detailed sample description**

Below we provide more details about the analysis of ten SSBs.

**X1 -** A calcareous SSB originating from the southern part of the cave (square G/21, elevation 715-720 below datum). The macro-traces observed on the SSB consist of negatives of micro-flakes localized on the high ridges and all around the tool, as well as of macro-striations. On this tool, use-wear developed on top of the previously formed patina, partially removing it. Smooth/flat micro-polishes appear on the high ridges. In several cases, these oriented polishes are partly abraded by new thrusting percussions that produce a rough topography as a result. No residue was identified.

**X2** - A calcareous SSB originating from the southern part of the cave (square F/22 elevation 735–740 below datum). The macro-traces consist of negatives of micro-flakes localized on the high ridges, and of macro-striations; flat surfaces with a specific orientation are also visible. On this tool, use wear developed on top of the previously formed patina, partially removing it. Residues consist of striated organic film as well as crushed compact and spongy bone tissues and are located on the high ridges of the SSB.

**X3 -** A calcareous SSB – a split half ball found in the area adjacent to the central hearth from the south (I/16c 570-580). The macro-traces consist of negatives of micro-flakes localized on the convex surface of the rounded high ridges and associated with flat and oriented polishes. On this tool, use-wear developed on top of the previously formed patina, partially removing it. A glossy and striated organic bone film is localized on the top of the high ridges of the tool. Spots of crushed amorphous white residues of fat and fibers were also identified all over the tool, sometime associated to the film.

**X4 -** Like the previous item, this limestone SSB is a split half-ball. The macro-traces observed on the sample consist of negatives of micro-flakes localized on the high ridges and around the tool as well as of macro-striations; flat surfaces with a specific orientation are also present. On this tool, use-wear developed on top of the previously formed patina, partially removing it. An organic film has also been identified on specific areas of the tool.

**X5 -** A flint SSB collected under the rock shelf of the cave (C/9 890-900). The macro-traces observed on the SSB consist of ridges that appear rounded. The traces are localized all around the surface of the tool. The micro-polishes are localized on the high ridges and show a smooth texture and domed topography. No residues were identified on this item.

**X6 -** A calcareous SSB collected from the southern part of the cave (H/21 655-660). The macro-traces observed on this sample consist of negatives of the flakes localized on the high ridges and all around the tool. Very few residues were observed on this SSB. They consist of crushed compact and spongy bone tissues distributed on the higher part of the dorsal side of the item.

**X7 -** A calcareous SSB split item originating from the southwest area (F/17c 715-720). The macro-traces observed on the sample consist of triangular negatives of micro-flakes localized on the high ridges, on the convex surfaces. On this tool, use-wear developed on top of the previously formed patina, partially removing it. Patches of micro-polishes characterized by smooth texture and flat topography are present together with striations. Polishes with smooth texture and domed topography are present on the high ridge. On this SSB, residues are abundant and consist of glossy and striated bone film as well as spots of crushed amorphous white residues mixed with fat and fibers. The residues are distributed on the high ridges. Crushed compact and spongy bone tissues mixed with greasy fat matter are abundant on the flat surface of the tool.

**X8** A calcareous SSB split item from the southwest area (E/17d 715-720). The macro-traces observed on the item consist of negatives of flakes localized on the high ridges, all around the item. On this tool, use-wear developed on top of the previously formed patina, partially removing it. Sheen appearance it is evident on the tool. The micro-polishes is localized on the top of high ridges, associated with long micro-striations and characterized by smooth texture and domed topography. Residues consist of glossy and striated organic bone film mostly localized on top of the high ridges, often in association with amorphous white fat residue. Spots of crushed compact and spongy bone tissues were identified on the tool’s scars.

**X9 -** A split half-ball calcareous SSB calcareous rock SSB (H/21 630-635). The macro-traces consist of negatives of micro-flakes localized on the high ridges and all around the tool, as well as macro-striations. On this tool, use-wear developed on top of the previously formed patina, partially removing it. Residues are not abundant and consist of crushed compact and spongy bone tissues localized on the flat surface of the SSB.

**X10** - A split half-ball calcareous SSB (G/21 670-675). The macro-traces observed on the sample consist in negatives of micro-flakes localized on the high ridges, all around the item, and in flat surfaces. On this tool, use-wear developed on top of the previously formed patina, partially removing it. Residues are not abundant and consist of glossy/striated organic bone film as well as spots of crushed white amorphous fat localized all over the item.

**Analysis of faunal results produced through the experimental trials**

The bones used in EXP-2 were initially cleaned by boiling them in a solution of water and neutral detergent for 2 hours. When more gestures were required in order to break the bone, most experimenters repeated the action on the same area, generating numerous small splinters (<5–10 mm). The remaining bone fragments from each series were collected and categorized according to individual, skeletal element, and SSB type used for the trial. Bone fragments longer than 3 cm (hardly any bone fragments of 2 cm were produced) were analyzed and classified according to criteria established by Villa and Mahieu [RS5]. The outline (transverse, curved/V-shape, longitudinal), fracture angle (oblique, right, mixed), and surface edge (smooth, jagged) were recorded along with the shaft circumference type and length (in millimetres). Surface damage, including percussion pits, percussion notches, and impact flakes produced during bone breakage, were also analysed [RS6].

Two to five minutes were required to fracture the bones, depending on the individual’s experience. Other constraints, such as the physical characteristics (such as strength) of the individual and his or her knowledge of bone anatomy and the morphology of the spheroid and anvil, also affect the processing time.

The percussion using SSBs produced a total of 73 fragments larger than 3 cm. Femurs produced 34 such splinters: 8 or 9 *per* individual. In contrast, the humeri showed a higher variability: 3 to 9 splinters per individual. The resulting bone fragments ranged from 20 mm in length in the case of humeri to 256 mm for femurs (Table S1).

A total of 322 fracture planes were analyzed, of which 89 (27.64%) were transversal, 125 (38.82%) were curved/V-shaped, and 108 (33.54%) were longitudinal. The fracture angles were mainly oblique (n= 131, 40.68%), and the resulting surfaces were smooth in 77.64% of the cases (n=250). The shaft fragments did not exceed ¼ of the circumference in 53.42% of the cases (n= 39), and in 30.14% of cases, the fragments exceeded ≥ ¾ of the circumference (n= 22) (Table S2).

The direct percussion produced different diagnostic elements: impact flakes (n=25, 34.25% of all fragments) and notches (n=35,47.9%). Among the latter, single notches predominated, characterized by fracture planes with smooth textures (n= 26, 74.3% of all notches). Three overlapped medullar negatives (8.6%) and six opposing notches (8.2%) were recorded in substantially smaller frequencies (Table 4). Notches occur preferably on longitudinal and curved bone outlines in femurs (n= 11, 84.6%) and on curved outlines in humeri (n= 16,72.7%). Eight percussion notches (22.9%) showed a typical large conchoidal scar on the medullar surface, ranging from 5 to 16 cm in length. However, this noteworthy phenomenon could be related to the total removal of the periosteum before breaking, because doing so would allow the expansion of the impact following the collagen lines. The periosteum is a membrane that covers the outer surface of all bones (except at the joints of long bones) and is composed of two strata: an outer "fibrous layer" and inner "cambium layer" (or "osteogenic layer"). This membrane protects the bone and dampens the blows made during fracturing to access the bone marrow. If this membrane is removed to facilitate bone breakage [e.g., RS7, RS8], the force of the blow can expand along the collagen lines, generating percussion notches with a larger diameter than when the periosteum remains attached to the bone.

**Table S1. Summary of the main characteristics and variables taken into account during the bone breakage process in each experimental series.**

| **Trial** | **Spheroid** | **Individual** | **Skeletal element** | **Processing time** | **No. Of fragments** | **Measuring range** | **No. Of breakage planes** | **Diagnostic features** | | | | |
| --- | --- | --- | --- | --- | --- | --- | --- | --- | --- | --- | --- | --- |
|  |  |  |  |  |  | **Length (mm)** |  | **OpN** | **OvN** | **SN** | **IF** | **PPits** |
| A | Sph 3 | Indiv 1 | Femur (right, cow) | 2' | 8 | 30-256 | 43 | - | - | 3 | 2 | 17 |
| B | Sph 3 | Indiv 1 | Femur (left, cow) | 1'55'' | 8 | 72-233 | 42 | - | - | 3 | 2 | 2 |
| C | Sph 2 | Indiv 1 | Femur (left, cow) | 2'13'' | 5 | 40-175 | 20 | 2 | - | 1 | 2 | 7 |
| D | Sph 2 | Indiv 5 | Humerus (right, cow) | 4'24'' | 6 | 20-182 | 23 | - | - | 1 | 2 | 8 |
| E | Sph 2 | Indiv 2 | Humerus (left, cow) | 3'38'' | 5 | 95-167 | 29 | 2 | - | 4 | 1 | 7 |
| F | Sph 1 | Indiv 3 | Humerus (left, cow) | 5'16'' | 7 | 20-170 | 28 | - | - | 4 | 2 | 40 |
| G | Sph 1 | Indiv 4 | Humerus (left, cow) | 4'11'' | 9 | 20-166 | 38 | 2 | 3 | 2 | 5 | 34 |
| H | Sph 1 | Indiv 1 | Femur (right, cow) | 2'15'' | 9 | 32-210 | 35 | - | - | 3 | 5 | 3 |
| I | Sph 2 | Indiv 1 | Femur (left, cow) | 3'02'' | 9 | 43-211 | 35 | - | - | 1 | 4 | 6 |
| J | Sph 6 | Indiv 1 | Humerus (right, sheep) | 2'17'' | 3 | 38-65 | 13 | - | - | 2 | - | - |
| K | Sph 2 | Indiv 1 | Radius-ulna (right, sheep) | 3'14'' | 4 | 35-90 | 16 | - | - | 2 | - | 1 |

OpN, opposing notches; OvN, overlapping notches; SN, single notch; IF, impact flakes; PPits, percussion pits.

**Table S2. Frequencies of fracture outlines, fracture angles, fracture edges, and shaft circumferences for limb bone fragments (≥ 3 cm).**

|  |  | **Femur** | % | **Humerus** | % | **Radius-Ulna** | % | **Total** | % |
| --- | --- | --- | --- | --- | --- | --- | --- | --- | --- |
| **No. Bone fragments** |  | 34 |  | 35 |  | 4 |  | 73 |  |
| **No. Breakage planes** |  | 151 |  | 155 |  | 16 |  | 322 |  |
| **Fracture outline** | Transverse (%) | 36 | 23,8 | 51 | 32,9 | 2 | 12,5 | 89 | 27,64 |
|  | Curved/V-shaped (%) | 63 | 41,7 | 54 | 34,84 | 8 | 50 | 125 | 38,82 |
|  | Longitudinal (%) | 52 | 34,4 | 50 | 32,26 | 6 | 37,5 | 108 | 33,54 |
| **Fracture angle** | Oblique (%) | 62 | 41,1 | 62 | 40 | 7 | 43,75 | 131 | 40,68 |
|  | Right (%) | 47 | 31,1 | 34 | 21,94 | 6 | 37,5 | 87 | 27,02 |
|  | Mixed (%) | 42 | 27,8 | 59 | 38,06 | 3 | 18,75 | 104 | 32,30 |
| **Fracture edge** | Smoothed (%) | 126 | 83,4 | 111 | 71,61 | 13 | 81,25 | 250 | 77,64 |
|  | Jagged (%) | 25 | 16,6 | 44 | 28,39 | 3 | 18,75 | 72 | 22,36 |
| **Bone circumference** | ≤ ¼ | 20 | 58,8 | 17 | 48,57 | 2 | 50 | 39 | 53,42 |
|  | 1/4-1/2 | 3 | 8,82 | 4 | 11,43 |  |  | 7 | 9,59 |
|  | 1/2-3/4 | 3 | 8,82 | 2 | 5,714 |  |  | 5 | 6,85 |
|  | ≥ ¾ | 8 | 23,5 | 12 | 34,29 | 2 | 50 | 22 | 30,14 |

**References**

1. Karkanas P, Shahack-Gross R, Ayalon A, Bar-Matthews M, Barkai R, Frumkin A, et al. Evidence for habitual use of fire at the end of the Lower Paleolithic: Site-formation processes at Qesem Cave, Israel. J Hum Evol. 2007; 53(2): 197-212.
2. Barkai R, Gopher A, On anachronism: The curious presence of Spheroids and Polyhedrons at Acheulo–Yabrudian Qesem Cave, Israel. Quat Int. 2016; 398: 118-28.
3. Blasco R, Rosell J, Sanudo P, Gopher A, Barkai R. What happens around a fire: faunal processing sequences and spatial distribution at Qesem Cave (300 ka), Israel. Quat Int. /2016; 398: 190-209.
4. Parush Y, Gopher A, Barkai R. Amudian versus Yabrudian under the rock shelf: A study of two lithic assemblages from Qesem Cave, Israel. Quat Int. 2016: 398: 13-36.‏
5. Villa P, Mahieu E. Breakage patterns of human long bones. J Hum Evol. 1991; 21(1): 27-48.
6. Pickering TR, Egeland CP. Experimental patterns of hammerstone percussion damage on bones: implications for inferences of carcass processing by humans. J Archaeol Sci. 2006; 33(4): 459-69.
7. Binford L.R. Bones: Ancient Men and Modern Myths.‏ Academic Press, San Diego, California. 1981.
8. Shipman P, Rose J. Early hominid hunting, butchering, and carcass-processing behaviors: approaches to the fossil record.  J. Anthropol. Archaeol. 1983; 2(1): 57-98.‏
